# IGF‐1 Deficiency Serves as an Integrated Biomarker Pathogenic Driver and Predictor in Poor Ovarian Response

**DOI:** 10.1002/advs.202514483

**Published:** 2026-01-29

**Authors:** Zhu Hu, Yuanyuan Yu, Guanyou Huang, Jinnan Li, Chao Yang, Aizhuan Long, Jia Tang, Tengxiang Chen, Shuyun Zhao, Tuo Zhang

**Affiliations:** ^1^ Reproductive Medicine Center the Affiliated Hospital of Guizhou Medical University Guiyang Guizhou China; ^2^ Transformation Engineering Research Center of Chronic Disease Diagnosis and Treatment Department of Physiology College of Basic Medicine Guizhou Medical University Guiyang Guizhou China

**Keywords:** assisted reproductive technology, granulosa cells, IGF‐1, poor ovarian response, prediction model

## Abstract

Poor ovarian response (POR) constitutes a notable clinical challenge within the domain of assisted reproductive technology, primarily attributable to the lack of reliable biomarkers for precise diagnosis and treatment. This study reveals significantly reduced levels of insulin‐like growth factor 1 (IGF‐1) in the serum, follicular fluid (FF), and granulosa cells (GCs) of patients with POR in comparison to those exhibiting a normal ovarian response (NOR). Notably, FF IGF‐1 concentrations demonstrated significant positive correlations with crucial IVF outcomes, including the numbers of metaphase II (MII) oocytes, 2‐pronuclear zygotes, and high‐quality embryos. To establish causality, we employed complementary in vivo models: systemic insulin‐like growth factor binding protein acid labile subunit (*Igfals*) knockout mice and granulosa cell specific IGF‐1 receptor (*Igf‐1r*) knockout mice. These models collectively demonstrated that disruption of the IGF‐1 signaling axis impairs follicle‐stimulating hormone (FSH) responsiveness and arrests follicular development at the secondary stage, thereby recapitulating the core POR phenotype. Building on these mechanistic insights, we developed novel clinical prediction tools based on FF IGF‐1: a POR risk model [Area under the curve (AUC) = 0.914] and a pregnancy outcome nomogram (AUC = 0.893), both of which significantly outperform traditional ovarian reserve parameters (such as anti‐Müllerian hormone and antral follicle count). Decision curve analysis (DCA) further validated a substantial clinical net benefit. This study aids clinicians in the early identification of patients with POR and provides a theoretical foundation for timely intervention and adjustment of treatment strategies.

## Graphical abstract

IGF‐1 deficiency underlies poor ovarian response (POR), as reduced levels in follicular fluid and granulosa cells impair antral follicle formation and compromise reproductive outcomes. Including IGF‐1 as a biomarker significantly enhances the accuracy of models predicting both PORrisk and pregnancy success.

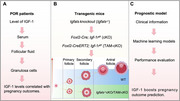

## 1 Introduction

The increasing prevalence of infertility has positioned assisted reproductive technology (ART) as an essential intervention [1]. The success of ART is largely dependent on controlled ovarian stimulation (COS) to procure multiple developmentally competent oocytes [2]. Nevertheless, a significant challenge is presented by poor ovarian response (POR), which affects between 5.6% and 35.1% of patients. It is characterized by a diminished follicle reserve and a reduced oocyte yield despite the administration of high doses of gonadotropin (Gn) [3]. Although standardized diagnostic criteria, such as the Bologna criteria, are available, the pathophysiological mechanisms underlying POR remain inadequately understood, thereby impeding the development of effective biomarkers and targeted treatments [4].

In 2016, the Poseidon group introduced a classification system that divides POR into four distinct subtypes: Groups 3 and 4 are associated with an anticipated diminished ovarian reserve, while Groups 1 and 2 maintain preserved ovarian function but demonstrate a suboptimal oocyte yield, termed “unexpected POR” [5, 6]. This classification underscores the limitations of traditional assessment systems, which rely on age, anti‐Müllerian hormone (AMH), and antral follicle count (AFC), in accurately identifying cases of unexpected POR [7, 8]. The prevailing therapeutic strategy, marked by substantial costs and limited effectiveness, imposes considerable financial burdens and contributes to a detrimental cycle of biological, psychological, and social health deterioration [3]. This cycle is sustained by recurrent treatment failures and the resultant anxiety and depression, underscoring the urgent need for the development of novel predictive biomarkers that facilitate early intervention.

IGF‐1, a crucial mediator of growth hormone (GH) signaling, has emerged as a significant regulator in folliculogenesis. It promotes GCs proliferation [9], steroidogenesis [10, 11], and oocyte maturation [12, 13] by activating the Phosphatidylinositol 3‐kinase/AKT and Mitogen‐activated protein kinase pathways through binding to the IGF‐1R [14]. Clinical studies consistently report that serum and FF IGF‐1 levels correlate with fetal growth parameters; higher IGF‐1 associates with increased neonatal weight and length, while insulin‐like growth factor‐binding protein 1 (IGFBP‐1, an IGF‐1 inhibitor) shows inverse relationships [15]. In pathological pregnancies, such as those complicated by gestational diabetes, reduced serum total IGF‐1 levels are predictive of adverse outcomes, including macrosomia or preterm birth, thereby highlighting its endocrine significance [16]. The role of IGF‐1 in the pathophysiology of POR, particularly its impact on folliculogenesis and subsequent pregnancy outcomes, remains inadequately investigated. This gap in understanding hinders the development of predictive models for personalized ART interventions.

## 2 Results

### 2.1 Serum Lower Total IGF‐1 Levels are Associated with Poor Ovarian Response and Inferior Reproductive Outcomes

**FIGURE 1:**
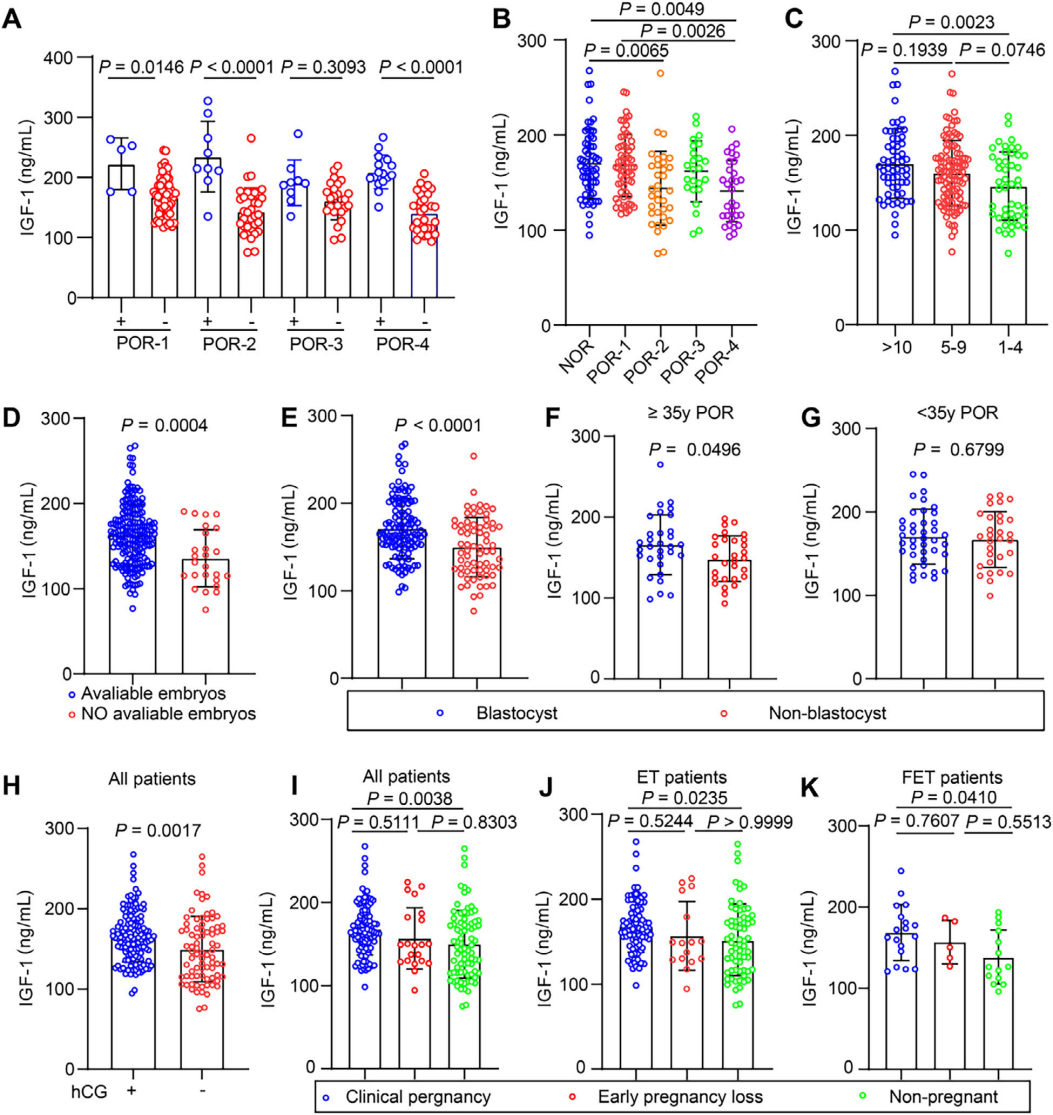
Serum total IGF‐1 levels and their correlation with clinical parameters in patients with different ovarian responses. (A) Serum total IGF‐1 levels in somatropin‐supplemented (+) versus un‐supplemented (‐) patients within each POR subgroup (POR‐1^+^, *n* = 5 vs POR‐1^−^, *n* = 61; POR‐2^+^, *n* = 9 vs POR‐2^−^, *n* = 34; POR‐3^+^, *n* = 9 vs POR‐3^−^, *n* = 24; POR‐4^+^, *n* = 16 vs POR‐4^−^, *n* = 31). *p*‐values are Bonferroni‐adjusted for four comparisons. (B) Serum total IGF‐1 levels in patients with a normal ovarian response (NOR, *n* = 58) versus untreated POR subgroups (POR‐1, *n* = 61; POR‐2, *n* = 34; POR‐3, *n* = 24; POR‐4, *n* = 31). The NOR cohort originally consisted of 60 patients; two were excluded due to somatropin administration during COS. (C) Serum total IGF‐1 levels stratified by oocyte yield (10–15, *n* = 58; 5–9, *n* = 104; 1–4, *n* = 46). (D) Comparison of serum total IGF‐1 levels between patients with (*n* = 183) and without (*n* = 25) available embryos in the entire untreated cohort (*n* = 208). (E) Among patients with available embryos (*n* = 183), comparison of serum total IGF‐1 levels between those who developed blastocysts (*n* = 115) and those with non‐blastocysts (*n* = 68). (F) Among POR patients aged <35 years, comparison of serum total IGF‐1 levels between those with blastocysts (*n* = 40) and those with non‐blastocysts (*n* = 30). (G) Among POR patients aged ≥ 35 years, comparison of serum total IGF‐1 levels between those with blastocysts (*n* = 31) and those with non‐blastocysts (*n* = 29). (H) Serum total IGF‐1 levels in the entire untreated cohort (*n* = 208) compared between patients with a positive (*n* = 128) and negative (*n* = 80) serum β‐hCG test. (I) Serum total IGF‐1 levels in the untreated cohort (*n* = 208) stratified by pregnancy outcome: clinical pregnancy (*n* = 107), early pregnancy loss (*n* = 21), or non‐pregnancy (*n* = 80). (J) Serum total IGF‐1 levels by pregnancy outcome after fresh embryo transfer (clinical pregnancy, *n* = 89; early pregnancy loss, *n* = 16; non‐pregnancy, *n* = 66; total *n* = 171). (K) Serum total IGF‐1 levels by pregnancy outcome after frozen‐thawed embryo transfer (clinical pregnancy, *n* = 18; early pregnancy loss, *n* = 5; non‐pregnancy, *n* = 14; total *n* = 37). For all panels, data are mean ± SEM. Specific *P*‐values and the statistical tests used for each comparison (*t*‐test, Mann–Whitney, ANOVA/Tukey, or Kruskal–Wallis/Dunn) are denoted directly on the figure panels.

Serum total IGF‐1 levels, measured on the day of human chorionic gonadotropin (hCG) trigger, were first evaluated for their association with clinical parameters in patients with POR and NOR (Table S1). Given that GH elevates serum IGF‐1 [17], we confirmed that somatropin users exhibited significantly higher IGF‐1 levels than non‐users within each subgroup (Figure 1A). Subsequent analyses were therefore adjusted for GH use. After excluding somatropin‐treated patients, serum total IGF‐1 levels were significantly decreased in the POR subgroups compared to NOR group, particularly POR‐2 and POR‐4 (Figure 1B). This positive correlation with oocyte yield was reinforced by the observation that patients yielding 1–4 oocytes had lower IGF‐1 levels than those with 5–9 or ≥ 10 oocytes (Figure 1C). Given this association with oocyte yield, we investigated whether IGF‐1 levels further correlated with embryonic development. Patients with no available embryos demonstrated significantly lower IGF‐1 levels than those with available embryos (Figure 1D). Moreover, among patients with available embryos, the failure to form blastocysts was associated with significantly lower IGF‐1 levels (Figure 1E). Age‐stratified analysis revealed that this association between low IGF‐1 and blastocyst formation failure was driven primarily by older patients (≥ 35 years); in this cohort, the non‐blastocyst group had significantly lower levels than the blastocyst group (Figure 1F). A similar, though non‐significant, trend was observed in younger patients (Figure 1G). We evaluated the association between IGF‐1 and early pregnancy outcomes. Patients with a positive β‐hCG test had significantly higher IGF‐1 levels than those with a negative result (Figure 1H). Serum total IGF‐1 levels were highest in the clinical pregnancy cohort, intermediate in the early pregnancy loss cohort, and lowest in the non‐pregnant cohort (Figure 1I). This pattern of higher IGF‐1 levels in clinical pregnancy held true regardless of the type of embryo transfer (Figure 1J,K). To quantitatively assess these observed associations, we performed regression analyses. Initial linear regression between serum total IGF‐1 and key continuous outcomes (total gonadotropin dose, oocyte yield, 2PN count, high‐quality embryo count) showed *P*‐values <0.05 but low coefficients of determination (R^2^ range: 0.02173–0.2169), suggesting weak linear correlations (Figure S1A–D). As these outcome variables are count data prone to over‐dispersion, we applied negative binomial regression, a more appropriate model. Under this model, serum total IGF‐1 level showed no statistically significant association with any of these outcomes (all *p* > 0.05). Notably, for high‐quality embryo count, the association approached but did not reach significance (*p* = 0.085; IRR = 1.157) (Figure S1E). Collectively, these results demonstrate that lower serum IGF‐1 levels are associated with poor ovarian response, impaired embryonic development, and unfavorable early pregnancy outcomes, while quantitative regression models did not reveal strong continuous associations with key laboratory parameters.

### 2.2 Deficient FF IGF‐1 Underlies POR and Predicts Embryological and Clinical Outcomes

**FIGURE 2:**
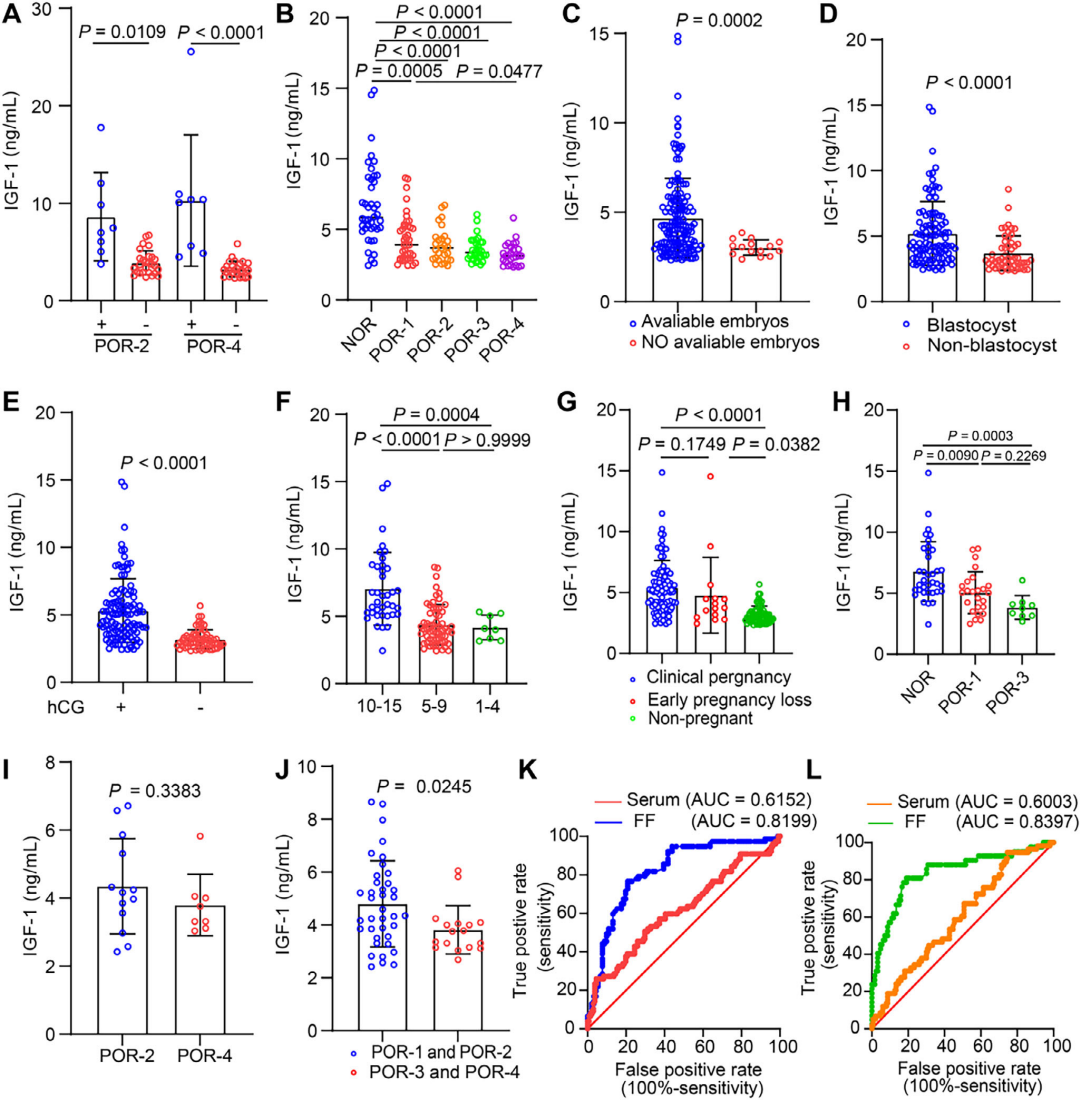
FF IGF‐1 levels associate with ovarian response and reproductive outcomes in IVF cycles. (A) FF IGF‐1 levels in somatropin‐supplemented (+) versus unsupplemented (‐) patients across POR subgroups. Planned comparisons (Bonferroni‐adjusted) were performed in POR‐2 (*n* = 8^+^ vs 27^−^) and POR‐4 (*n* = 8^+^ vs 27^−^). Data for POR‐1 (2^+^ vs 41^−^) and POR‐3 (4^+^ vs 31^−^) are shown descriptively. (B) FF IGF‐1 levels in untreated normal responders (NOR, *n* = 42) versus POR subgroups (POR‐1, *n* = 41; POR‐2, *n* = 27; POR‐3, *n* = 31; POR‐4, *n* = 27). (C) FF IGF‐1 levels in patients with (*n* = 153) versus without (*n* = 15) transferable embryos. (D) Association between FF IGF‐1 levels and usable blastocyst formation among patients with transferable embryos (with blastocysts, *n* = 99; without, *n* = 54). (E) FF IGF‐1 levels in patients with positive (*n* = 106) versus negative (*n* = 62) serum hCG outcomes. (F) FF IGF‐1 levels in hCG‐positive patients stratified by oocyte yield (Number of oocytes: 10–15, *n* = 38; 5–9, *n* = 60; 1–4, *n* = 8). (G) FF IGF‐1 levels by pregnancy outcome after embryo transfer (clinical pregnancy, *n* = 91; early pregnancy loss, *n* = 15; non‐pregnant, *n* = 62). (H) FF IGF‐1 levels in young patients (< 35 years) with clinical pregnancy, stratified by response type (NOR, *n* = 36; POR‐1, *n* = 25; POR‐3, *n* = 9). (I) FF IGF‐1 levels in older patients (≥ 35 years) with clinical pregnancy, comparing POR‐2 (*n* = 13) and POR‐4 (*n* = 8) subgroups. (J) FF IGF‐1 levels in poor responders with clinical pregnancy, comparing young (< 35 years, *n* = 38) and older (≥ 35 years, *n* = 17) patients. (K) ROC curves comparing the predictive accuracy of FF IGF‐1 (AUC = 0.8199; *n* = 168) versus serum IGF‐1 (AUC = 0.6152; *n* = 208) for clinical pregnancy in POR patients (DeLong's test, *p* < 0.0001). (L) ROC curves comparing FF IGF‐1 (AUC = 0.8397; *n* = 168) versus serum IGF‐1 (AUC = 0.6003; *n* = 208) for POR diagnosis (DeLong's test, *p* < 0.0001). Data are presented as box‐and‐whisker plots unless otherwise noted. Specific p‐values and statistical tests (*t*‐test, Mann–Whitney, Kruskal–Wallis/Dunn) are indicated on the panels.

Given the pivotal role of FF in shaping the oocyte microenvironment and regulating follicular developmental potential [18, 19, 20], we quantified IGF‐1 levels in the FF from the first dominant follicle retrieved in POR patients (Table S2). Due to sample size limitations, analysis was restricted to POR‐2 and POR‐4 subgroups. Consistent with systemic findings, FF IGF‐1 levels were significantly higher in patients receiving somatropin pretreatment compared to non‐treated counterparts (Figure 2A). Critically, after excluding somatropin‐treated subjects to isolate the pathophysiology of POR, we discovered a pervasive IGF‐1 deficiency within the follicular microenvironment, with all POR subgroups exhibiting significantly lower FF IGF‐1 concentrations than the NOR controls (Figure 2B). FF IGF‐1 levels were lower in patients without available embryos compared to those with embryos (Figure 2C). Critically, NOR had more high‐quality embryos than all POR subgroups (Figure S2A). Furthermore, among patients with embryos, those who failed blastocyst formation had lower FF IGF‐1 levels than those who succeeded (Figure 2D). To account for age‐related confounding, age‐stratified analysis revealed no significant differences in IGF‐1 levels between patients with and without blastocyst formation in POR‐1 and POR‐3 subgroups aged <35 years (Figure S2B). However, in older POR‐2 and POR‐4 groups, IGF‐1 levels were significantly lower in those without blastocyst formation (Figure S2C). Regarding pregnancy outcomes, FF IGF‐1 levels were significantly higher in serum hCG‐positive patients at 14 days post‐embryo transfer compared to serum hCG‐negative patients (Figure 2E). To address potential confounding by oocyte yield, hCG‐positive patients were divided into three oocyte count groups. Among these patients, those with 10–15 oocytes had higher IGF‐1 levels than those with 5–9 or 1–4 oocytes, who showed no difference (Figure 2F). When categorized by pregnancy outcome, FF IGF‐1 levels were highest in the clinical pregnancy group, followed by the early pregnancy loss group, and were lowest in the non‐pregnant group (Figure 2G). Among clinical pregnancy patients under 35, the NOR group had higher FF IGF‐1 levels than the POR‐1 and POR‐3 groups, with no difference between the latter two (Figure 2H); For patients 35 and older, no significant difference was found between the POR‐2 and POR‐4 groups (Figure 2I). Analysis of FF IGF‐1 levels in patients who achieved a clinical pregnancy demonstrated that levels were significantly lower in those with expected POR (subgroups POR‐3 and POR‐4) compared to those with unexpected POR (subgroups POR‐1 and POR‐2) (Figure 2J). Negative binomial regression demonstrated that elevated FF IGF‐1 concentrations positively correlated with key embryological outcomes: the number of high‐quality embryos (IRR 1.212; *p* < 0.0001), MII oocytes (IRR 1.131; *p* = 0.0001), two‐Pronuclear Zygote (2PN) (IRR 1.152; *p* < 0.0001), and total oocytes retrieved (IRR 1.111; *p* = 0.005), but revealed no significant associations with either total gonadotropin dose or duration of gonadotropin stimulation (Table S3). Finally, we validated the clinical utility of IGF‐1 measurement. Receiver operating characteristic (ROC) curve analysis confirmed that both serum and FF IGF‐1 levels served as effective diagnostic and prognostic biomarkers, capable of identifying POR patients and predicting clinical pregnancy (Figure 2K,L). Collectively, these results position FF IGF‐1 not merely as a correlate but as a robust biomarker of multidimensional reproductive competence, integral to the oocyte microenvironment and offering significant diagnostic and prognostic utility.

### 2.3 Downregulation of IGF‐1 and IGF‐1R in GCs is Associated with POR

**FIGURE 3:**
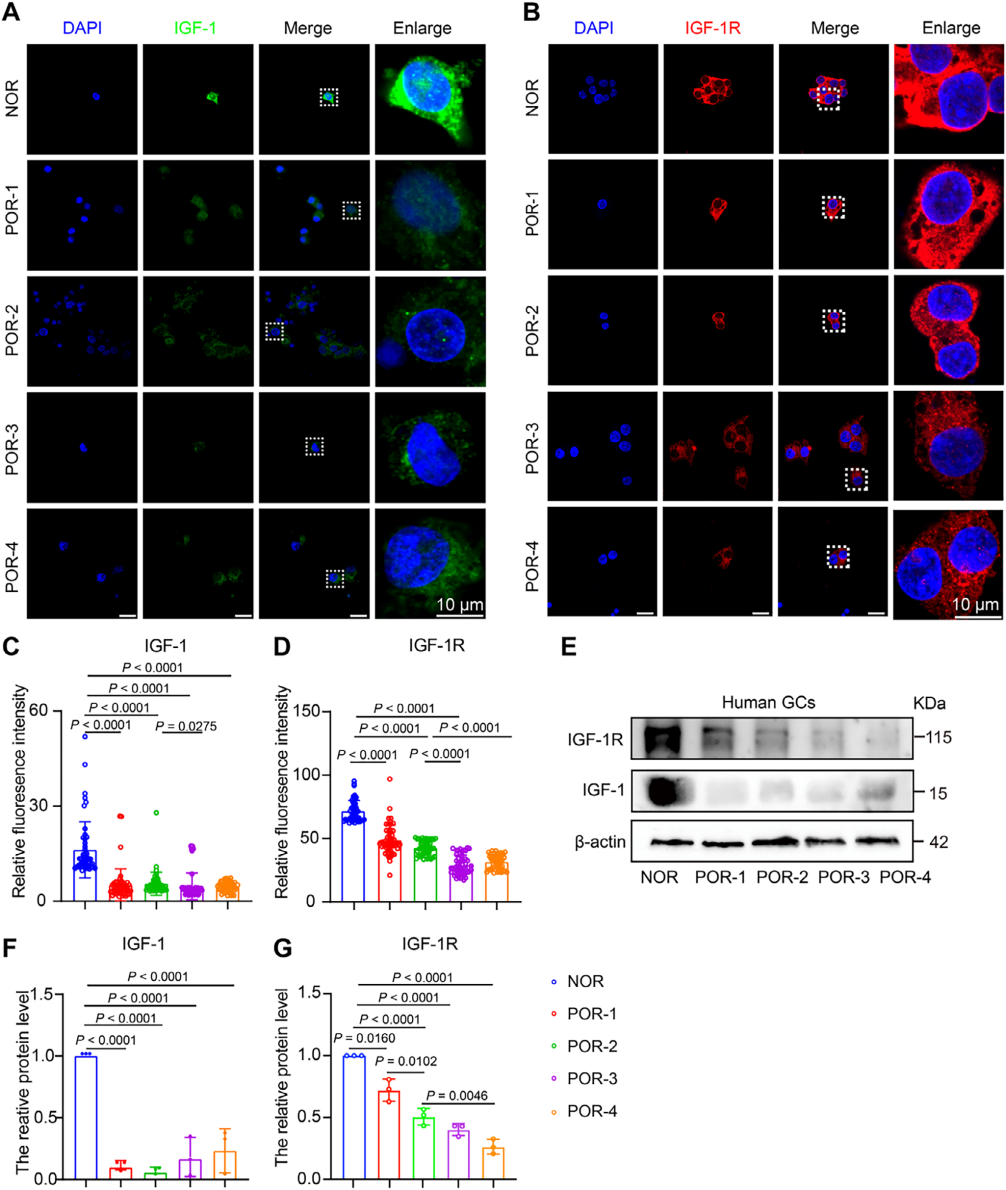
GCs IGF‐1/IGF‐1R expression is impaired in poor ovarian responders. (A) Representative IF images showing IGF‐1 (green) in GCs from NOR (*n* = 5 patients) and POR (*n* = 3 patients per subgroup) groups. Nuclei are stained with DAPI (blue). Scale bar: 10 µm. (B) Representative IF images of IGF‐1R (red) in GCs from the same patient cohorts. Scale bar: 10 µm. (C) Quantification of IGF‐1 fluorescence intensity in GCs (> 50 cells per group, ten fields per sample). (D) Quantification of IGF‐1R fluorescence intensity in GCs (> 50 cells per group, ten fields per sample). (E) Representative immunoblots of IGF‐1 and IGF‐1R protein expression in granulosa cell lysates from NOR and POR subgroups (*n* = 3 independent samples per group). (F,G) Relative IGF‐1 and IGF‐1R protein expression in GCs from NOR and POR subgroups (*n* = 3 per group). Data in C, D, F, and G are presented as mean ± SEM. Statistical comparisons versus the NOR group were performed using one‐way ANOVA with Dunnett's post‐hoc test. Specific *p*‐values are indicated on the graphs.

GCs critically support oocyte maturation through metabolic provision, hormonal signal mediation, and structural integrity maintenance, essential processes for folliculogenesis [21, 22], whereas their functional alterations specifically regarding IGF‐1 expression in POR, remain incompletely defined. To explore their involvement in POR pathogenesis, GCs isolated from POR type 1–4 patients underwent IGF‐1 and IGF‐1R quantification. Immunofluorescence and Western blot analyses consistently revealed significantly diminished IGF‐1 and IGF‐1R protein levels compared to NOR (Figure 3A–G). This coordinated deficiency in GCs defines a core impairment in the intrafollicular IGF‐1 signaling pathway in POR.

### 2.4 Systemic IGF‐1 Deficiency Induced by *Igfals* Deletion Recapitulates Key Features of POR

**FIGURE 4:**
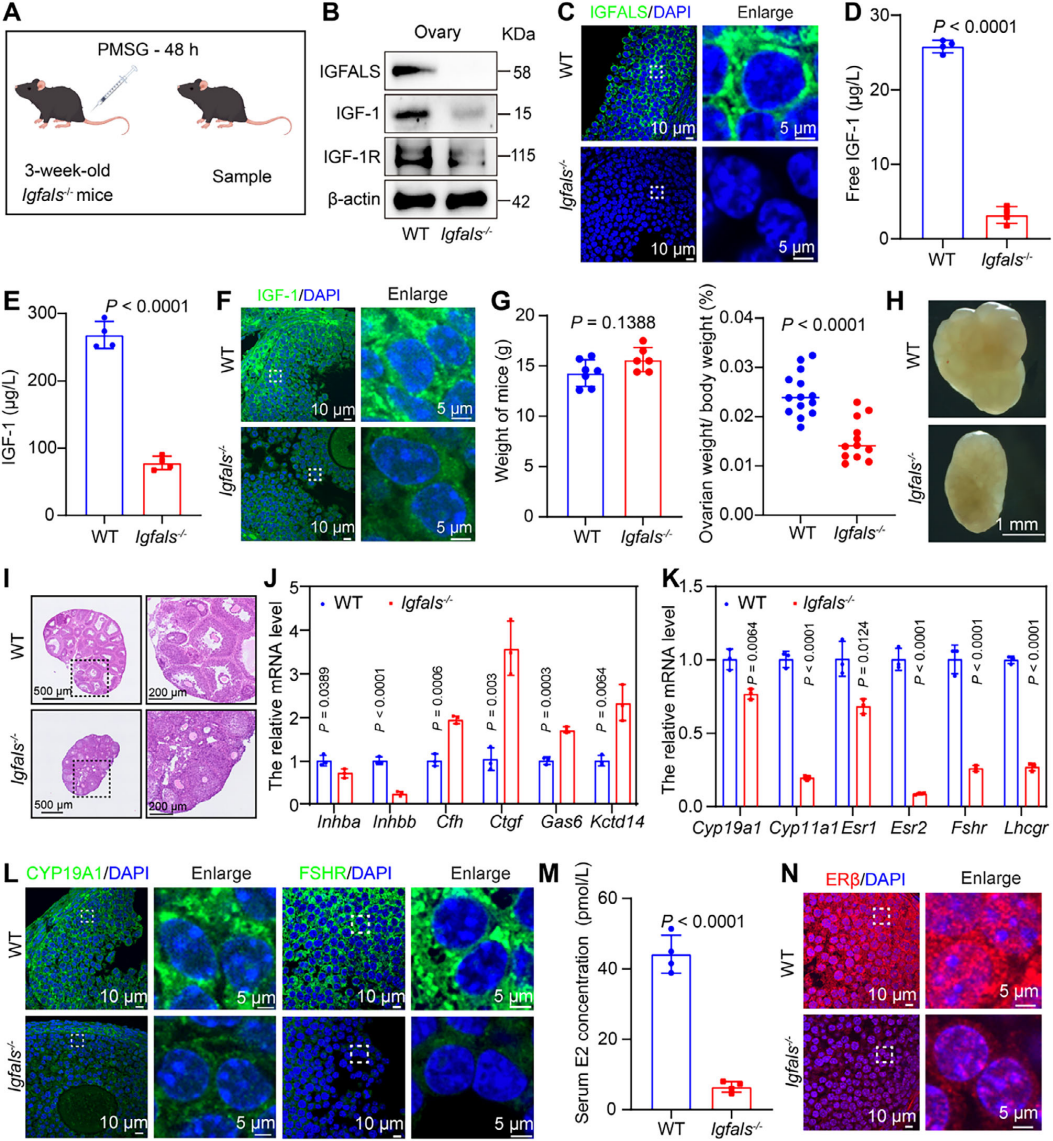
*Igfals* deletion impairs gonadotropin‐induced ovarian response via disruption of IGF‐1 signaling and follicular development. (A) Experimental timeline: 3‐week‐old WT and *Igfals ^−/−^* mice received 5 IU PMSG; ovaries and other tissues were collected 48 h later. (B) Representative Western blot analysis of ovarian tissues from WT and *Igfals^−/−^* mice demonstrates the ablation of IGFALS and reveals concomitant alterations in the protein levels of IGF‐1 and IGF‐1R. β‐actin is shown as a loading control. (C) Representative IF image of IGFALS (green) in ovarian sections from WT and *Igfals^−/−^* mice. DAPI (blue) was used as a nuclear counterstain. Scale bars are indicated. (D) Serum levels of free IGF‐1 in WT and *Igfals^−/−^* mice at 48 h following PMSG administration (*n* = 4 per group). (E) Serum levels of IGF‐1 in WT and *Igfals ^−/−^* mice 48 h after PMSG (*n* = 4 per group). (F) Representative IF image of IGF‐1 (green) in ovarian sections from WT and *Igfals^−/−^* mice. DAPI (blue) was used as a nuclear counterstain. Scale bars are indicated. (G) Body weight and ovary‐to‐body weight ratio in WT (*n* = 7) and *Igfals ^−/−^* (*n* = 6) mice. (H) Macroscopic view of ovaries from WT and *Igfals ^−/−^* mice. (I) Representative HE‐stained ovarian sections. (J) mRNA levels of follicular development markers: antral follicle (*Inhba, Inhbb*), preantral follicle (*Kctd14, Gas6*), and atretic follicle (*Ctgf, Cfh*) markers. (K) qPCR analysis of FSH‐responsive gene expression in WT and *Igfals ^−/−^* ovaries. (L) IF of ovarian sections stained for CYP19A1 (green), FSHR (green) with DAPI (blue). Scale bars indicated. (M) Serum levels of E2 in WT and *Igfals ^−/−^* mice 48 h after PMSG (*n* = 4 per group). (N) Representative IF images of ERβ (red) in ovarian sections from WT and *Igfals^−/−^* mice. Nuclei were counterstained with DAPI (blue). Scale bars are indicated. Panels D, E, G, J, K, and M present data as mean ± SEM. Statistical comparisons between the WT and *Igfals ^−/−^* groups for each experiment were performed using an unpaired, two‐tailed Student's *t*‐test.

To directly examine the pathophysiological implications of diminished IGF‐1 bioavailability in POR, we utilized a mouse model with a constitutive deletion of the *Igfals* gene (Figure 4A). This gene encodes the acid‐labile subunit, a protein essential for stabilizing the circulating IGF‐1 ternary complex and extending its half‐life [23]. First, Western blot analysis of multiple organ tissues revealed that, compared to wild‐type (WT) mice, *Igfals^−/−^* mice exhibited significantly reduced or nearly absent IGFALS protein expression in the heart, liver, spleen, lung, kidney, and ovarian tissues (Figure 4B; Figure S3A,B). IF staining and quantitative analysis showed the disappearance of the IGFALS fluorescent signal in ovarian tissue (Figure 4C; Figure S3C). Collectively, these results confirm the effective knockout of the *Igfals* gene both systemically and locally within the ovary. ELISA analysis demonstrated that the serum total IGF‐1 and free IGF‐1 level was significantly lower in *Igfals^−/−^* mice than in WT controls (Figure 4D,E), consistent with the core function of IGFALS in stabilizing the ternary complex and prolonging the half‐life of IGF‐1. Analysis of ovarian tissue revealed a significant downregulation in the protein expression of both IGF‐1 and its receptor, IGF‐1R, in *Igfals^−/−^* mice (Figure 4B; Figure S3B). This indicates a severe suppression of the local IGF‐1 signaling pathway in the ovary.

Notably, an important organ‐specific compensatory phenomenon was observed. While the protein expression of IGF‐1 and IGF‐1R showed varying degrees of reduction in the heart, spleen, lung, kidney, and ovary of *Igfals^−/−^* mice, their levels were significantly upregulated in the liver (Figure S3A,B). As the primary site of IGF‐1 synthesis, this hepatic compensatory response likely aims to counteract the rapid clearance of circulating IGF‐1 caused by ternary complex destabilization, attempting to maintain a baseline supply. This finding underscores the high dependency of peripheral organs like the ovary on circulatory IGF‐1. When the systemic delivery system fails, the ovary itself cannot effectively compensate, leading to local signal failure.

To determine the direct impact of systemic IGFALS deficiency on ovarian development, a systematic analysis was conducted on ovaries from 3‐week‐old mice treated with PMSG. Morphological observation showed that although there was no significant difference in body weight between *Igfals^−/−^* and WT mice, the absolute ovarian weight and ovary‐to‐body weight ratio were significantly reduced in *Igfals^−/−^* mice (Figure 4G). Bright‐field images clearly revealed that the ovarian volume of *Igfals^−/−^* mice was markedly smaller than that of WT controls (Figure 4H). HE staining showed that WT ovaries contained follicles at various developmental stages, including structurally intact preantral and antral follicles. In contrast, *Igfals^−/−^* ovaries completely lacked antral follicle structures, with follicular development universally arrested at the preantral stage (Figure 4I). qPCR analysis demonstrated that the expression of key antral follicle markers, specifically Inhibin beta A (*Inhba*) and Inhibin beta B (*Inhbb*), was significantly downregulated in *Igfals^−/−^* ovaries. In contrast, markers associated with preantral follicles, namely Potassium channel tetramerization domain containing 14 (*Kctd14*) and Growth arrest‐specific 6 (*Gas6*), along with markers of atretic follicles [24], Connective tissue growth factor (*Ctgf*) and Complement factor H (*Cfh*), were markedly upregulated (Figure 4J). These data demonstrate that follicular development in *Igfals^−/−^* mice is arrested at the critical transition point from the preantral to the antral stage, accompanied by an increased tendency toward atresia.

The follicle‐stimulating hormone signaling pathway is a key driver of follicular growth. To investigate whether impairment of the IGF‐1 signaling system affects the ovarian response to Gn, the expression of downstream FSH target genes was analyzed by qPCR in ovaries from PMSG‐stimulated mice. The results showed that, compared to WT mice, *Igfals^−/−^* mice exhibited significant suppression in the transcriptional levels of a series of key FSH‐responsive genes in the ovary. This included the FSH receptor gene (*Fshr*), the luteinizing hormone/chorionic Gn receptor gene (*Lhcgr*), and key steroidogenic enzyme genes (*Cyp19a1*, *Cyp11a1*). The expression of estrogen receptor genes (*Esr1*, *Esr2*) was also significantly downregulated (Figure 4K).

IF staining and quantitative analysis corroborated these gene expression changes. The protein expression intensity of FSHR, CYP19A1 (aromatase) in ovarian GCs was significantly weaker in *Igfals^−/−^* mice than in WT mice (Figure 4L; Figure S3C). Furthermore, ELISA detected a significant reduction in peripheral blood estradiol (E2) levels in *Igfals^−/−^* mice (Figure 4M). CYP19A1 is the rate‐limiting enzyme that catalyzes the conversion of androgens to estrogens; its downregulation is directly linked to the observed reduction in blood E2 levels (Figure 4L), and the expression of the estrogen receptor ERβ was decreased (Figure 4N; Figure S3C). These results indicate that the attenuation of IGF‐1 signaling caused by systemic IGFALS deficiency severely compromises the responsiveness of ovarian GCs to FSH stimulation.

### 2.5 Somatropin Adjunct Therapy Improves IVF Outcomes in POR and Acts via the IGF‐1/IGF‐1R Axis

**FIGURE 5:**
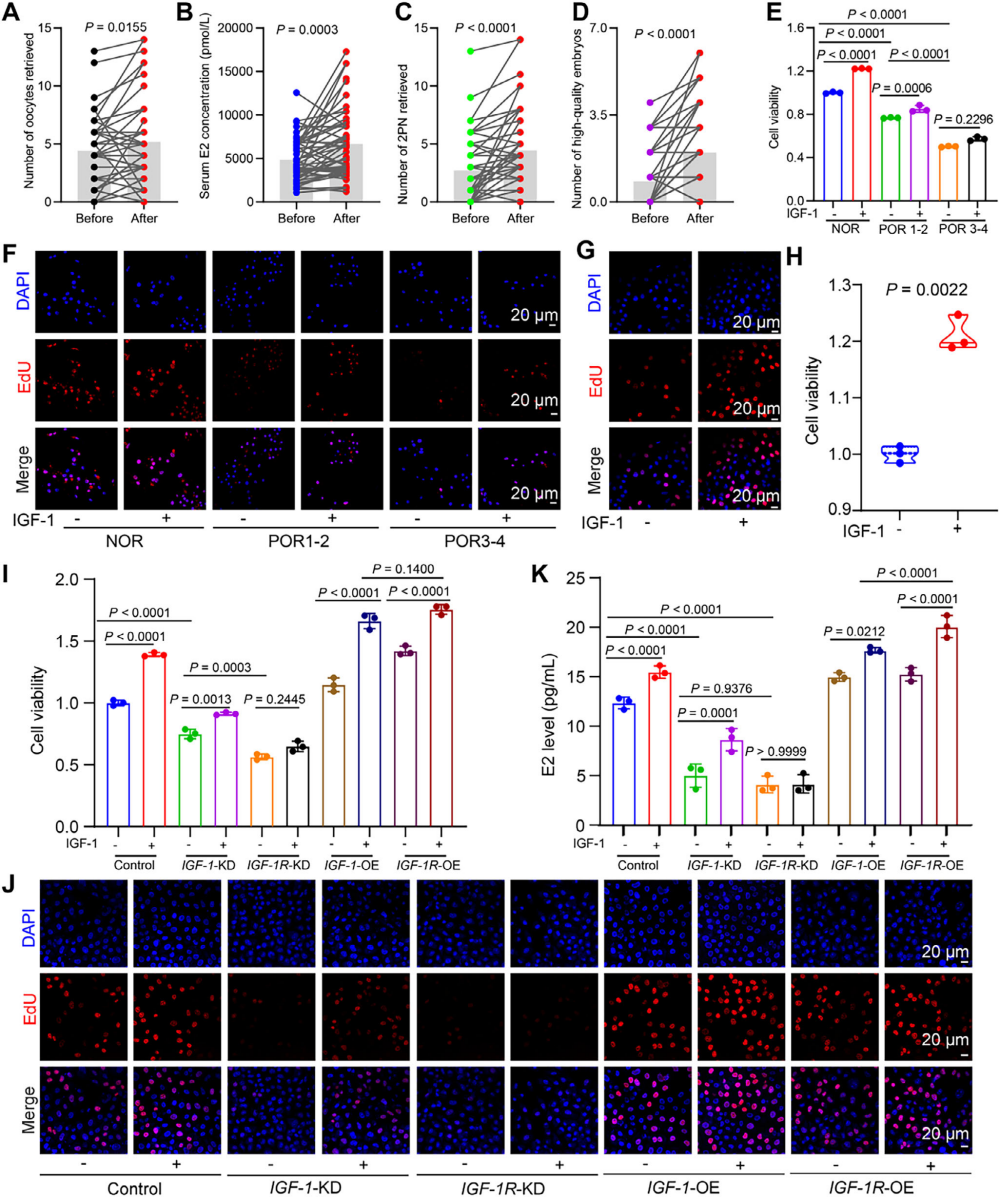
Somatropin supplementation improves IVF outcomes and IGF‐1 signaling promotes granulosa cell proliferation. (A–D) Self‐paired analysis of IVF outcomes in patients (*n* = 50) undergoing cycles with versus without somatropin supplementation. (A) Number of oocytes retrieved. (B) Serum E2 levels on hCG trigger day. (C) Number of 2PN zygotes. (D) Number of high‐quality embryos. (E) Proliferation of human GCs from NOR, POR1‐2, and POR3‐4 patients treated with or without 60 ng/mL recombinant lGF1 protein, assessed by CCK‐8 assay. Due to the limited availability of GCs from patients with POR, the POR subgroups were pooled into two groups: POR1‐2 and POR3‐4 (*n* = 3 independent biological replicates per group). (F) Representative EdU staining of human GCs from NOR and POR patients cultured with or without recombinant lGF1 protein (60 ng/mL). Proliferating cells (EdU, red); nuclei (DAPI, blue). Scale bar: 20 µm. (G) (H) EdU staining of mouse GCs treated with 0, 60 ng/mL recombinant lGF1 protein. Scale bar: 20 µm. (H) Proliferation of mouse primary GCs treated with recombinant lGF1 protein (60 ng/mL), measured by CCK‐8 assay (*n* = 3). (I) Proliferation of *IGF*‐1‐KD, *IGF*‐1‐OE, *IGF‐1R*‐KD, and *IGF‐1R*‐OE KGN cells after recombinant lGF1 protein (60 ng/mL) treatment, assessed by CCK‐8 assay (*n* = 3). (J) EdU staining of control and engineered KGN cells with or without recombinant lGF1 protein (60 ng/mL). Scale bar: 20 µm. (K) E2 secretion measured by ELISA in conditioned medium from the indicated cell lines after IGF‐1 treatment (n = 3). Data are presented as mean ± SD. Statistical significance was determined using a paired t‐test (A–D), an unpaired *t*‐test (H), or a two‐way ANOVA followed by Šidák's multiple comparisons test (E,(I–K).

To rigorously evaluate the potential therapeutic impact of somatropin (recombinant human growth hormone) in patients with predicted POR, we conducted a secondary analysis on a cohort previously excluded from our initial serum and FF studies due to concurrent somatropin treatment during their controlled ovarian stimulation cycles. This investigation employed a stringent within‐subject paired design, where each eligible POR patient contributed data from two IVF cycles performed at our center: one cycle supplemented with somatropin and one without. This methodological approach, by comparing critical outcome parameters within the same individual, effectively minimized confounding inter‐patient variables, allowing for a more precise attribution of observed effects to somatropin administration. Our findings revealed that cycles supplemented with somatropin exhibited statistically significant improvements across several key IVF laboratory and clinical parameters compared to the untreated control cycles from the same patients. Specifically, the mean number of oocytes retrieved and the peak serum estradiol level on the day of hCG trigger were significantly higher in the somatropin‐treated cycles (Figure 5A,B). More importantly, key indicators of embryonic developmental potential, the number of 2PN zygotes and the yield of high‐quality embryos, were also significantly increased following somatropin treatment (Figure 5C,D). These data collectively indicate that adjunctive somatropin therapy may positively influence IVF cycle outcomes in this difficult‐to‐treat POR subgroup.

Concurrently, biochemical analysis of paired samples from the same patients identified a critical associated phenomenon: somatropin treatment led to a synchronous and significant elevation in total IGF‐1 concentrations in both peripheral serum and FF (Figure S4A,B). This finding aligns with the classic endocrine mechanism of growth hormone action, whereby it stimulates the synthesis and secretion of endogenous IGF‐1, primarily from the liver, following activation of the growth hormone receptor [9]. This result directly links the observed clinical benefit of somatropin to its activation of the GH‐IGF‐1 endocrine axis.

The specific mechanistic role of the IGF‐1 signaling axis in regulating granulosa cell function were investigated. In primarily cultured human GCs, stimulation with exogenous recombinant IGF‐1 protein significantly promoted the proliferation of GCs derived from NOR and patients with the POR‐1/2 subtype. However, this pro‐proliferative effect was markedly attenuated in GCs isolated from patients with the POR‐3/4 subtype, which typically indicates a more severe diminution of ovarian reserve (Figure 5E). Consistently, under basal conditions (without IGF‐1 stimulation), GCs from patients with POR exhibited significantly lower proliferation compared to those from the NOR group. Notably, following IGF‐1 stimulation, proliferation was enhanced in both groups; however, the proliferative capacity of POR‐derived GCs remained significantly lower than that of the NOR group (Figure 5F; Figure S4C). This phenomenon of impaired IGF‐1 responsiveness was recapitulated and validated in parallel experiments using mouse primary GCs (Figure 5G,H; Figure S4D), suggesting it may be a conserved cellular phenotype of POR across species.

To precisely dissect the operational logic of the IGF‐1 signaling pathway at a genetic level, we successfully established four stable KGN human granulosa cell line variants using lentiviral transduction: *IGF‐1* knockdown (*IGF‐1‐*KD), *IGF‐1R*‐KD, IGF‐1 overexpression (*IGF‐1*‐OE), and *IGF‐1R*‐OE. Successful modulation was confirmed by qPCR and Western Blot (Figure S4E–G). Strikingly, Western blot analysis revealed a consistent co‐regulatory pattern: IGF‐1R protein baselines were downregulated in *IGF‐1* KD cells, while IGF‐1 protein levels decreased in *IGF‐1R* KD cells. Conversely, overexpression of either IGF‐1 or IGF‐1R synergistically upregulated the expression of the other molecule (Figure S4H,I). This “co‐varying” expression pattern between the ligand and its receptor closely mirrors our previous observations in primary GCs from POR patients and in ovarian tissues from systemic *Igfals^−/−^* knockout mice. Under conditions mimicking the in vivo antra follicle formation (with added FSH), we systematically evaluated the functional phenotypes of these engineered cell lines. Assessments of proliferative capacity (CCK‐8 assay) and EdU incorporation staining demonstrated that knockdown of either *IGF‐1* or *IGF‐1R* significantly inhibited cell proliferation compared to the control group. Critically, the inhibition caused by *IGF‐1R*‐KD was significantly more pronounced than that caused by *IGF‐1*‐KD (Figure 5I,J; Figure S4J). Conversely, overexpression of either molecule promoted proliferation, with the pro‐proliferative effect of *IGF‐1R‐*OE being significantly stronger than that of *IGF‐1*‐OE (Figure 5I,J; Figure S4J). The core endocrine function of GCs, measured by E2 secretion into the culture supernatant, showed perfectly parallel trends: knockdown reduced E2 secretion, overexpression increased it, and the effects of modulating IGF‐1R were consistently more potent than those of modulating IGF‐1 (Figure 5K).

To distinguish between pathological mechanisms rooted in “ligand deficiency” versus “impaired signal reception capability,” we performed rescue experiments by supplementing all genetically modified cell lines with exogenous IGF‐1 protein. The results showed that exogenous IGF‐1 could further enhance the viability and estrogen‐secreting capacity of control and *IGF‐1‐*KD cells. However, in *IGF‐1R*‐KD cells, even stimulation with a high concentration of exogenous IGF‐1 resulted in only marginal functional improvement, completely failing to restore cellular function to the baseline level of the control group (Figure 5I,J). This key evidence directly demonstrates that a severe deficiency in cell‐surface IGF‐1R leads to a fundamental breakdown of the entire IGF‐1 signaling pathway at the receptor level, rendering it incapable of effectively initiating downstream signal transduction cascades upon ligand supplementation. This finding elucidates the pivotal hub role of IGF‐1R from a cellular functional perspective.

### 2.6 GCs‐specific *Igf‐1r* Knockout Exhibits Pathological Features of POR

**FIGURE 6:**
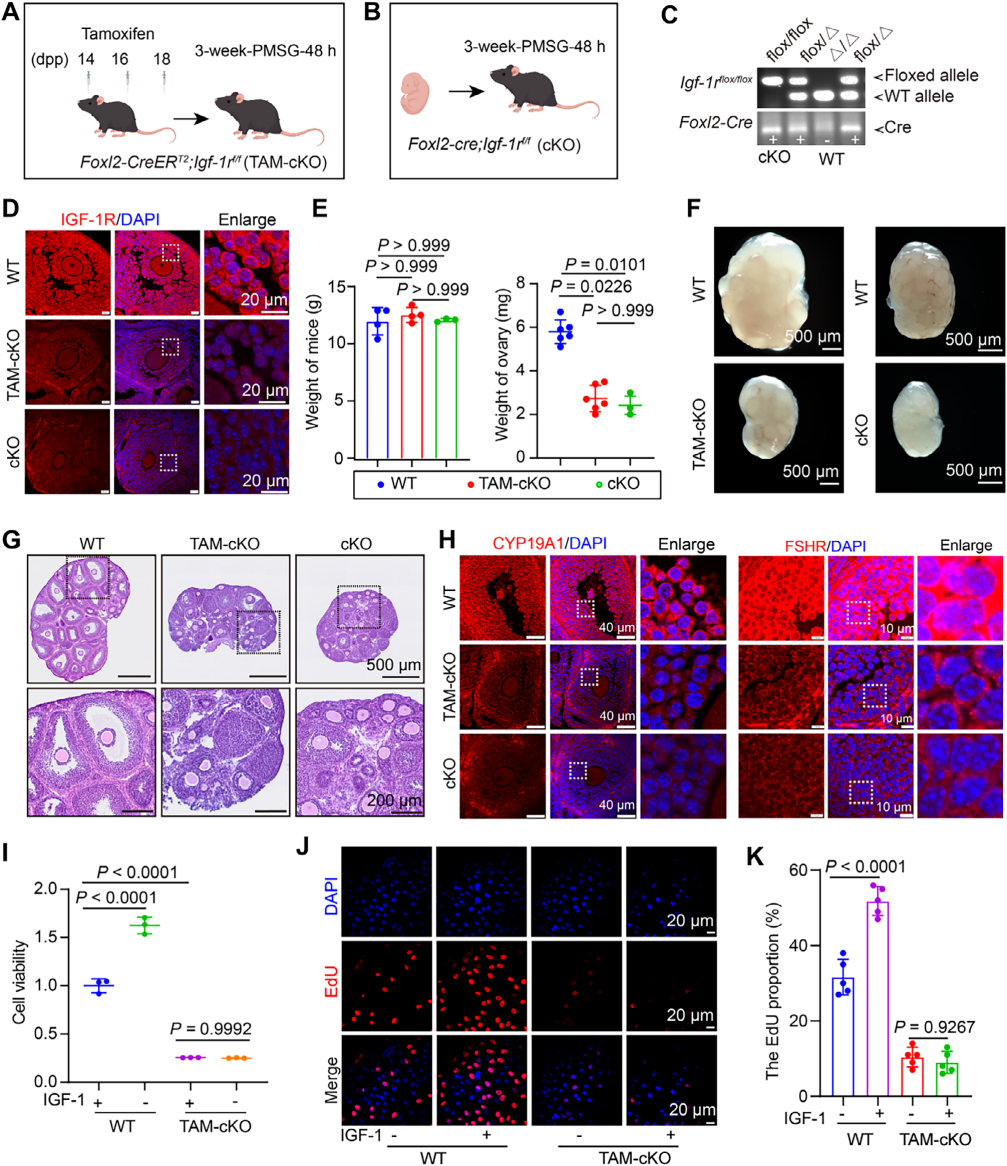
Granulosa cell specific deletion of *Igf‐1r* disrupts follicular development and IGF‐1 responsiveness. (A) Schematic of the inducible *Igf‐1r* knockout model (*Igf‐1r^flox/flox^; Foxl2‐CreER^T2^*; TAM‐cKO). (B) Schematic of the constitutive *Igf‐1r* knockout model *(Igf‐1r^flox/flox^*; *Foxl2‐Cre;* cKO), with deletion initiated by embryonic day 12.5. (C) Representative genotyping gel. (D) IF validation of IGF‐1R depletion in GCs (red: IGF‐1R; blue: DAPI). (E) Body and ovarian weights of 3‐week‐old WT, TAM‐cKO, and cKO mice measured 48 h after injection with 5 IU of PMSG (*n* = 4 per group). (F) Gross ovarian morphology. Scale bar: 500 µm. (G) HE‐stained ovarian sections. Scale bar: 500 µm. (H) Representative immunofluorescence images of CYP19A1 and FSHR (red) in ovarian sections. Nuclei are counterstained with DAPI (blue). (I) Proliferation of primary GCs from WT and TAM‐cKO mice treated with or without 60 ng/mL recombinant lGF1 protein, assessed by CCK‐8 assay over time (*n* = 3). (J) EdU incorporation in primary GCs from WT and TAM‐cKO mice (EdU, red; DAPI, blue). Scale bar: 20 µm. (K) Quantification of EdU‐positive GCs from (J). Data are presented as the mean percentage of EdU‐positive cells ± SEM from *n* = 3 independent experiments by an unpaired two‐tailed Student's *t*‐test. Data are presented as mean ± SEM. Statistical significance was determined by one‐way ANOVA with Tukey's post‐hoc test for the three‐group comparison in (E), and by two‐way ANOVA with Šidák's multiple comparisons test for the factors of genotype and IGF‐1 treatment in (I,K).

IGF‐1R, a high‐affinity tyrosine kinase receptor, initiates multiple downstream signaling pathways upon binding with its ligand IGF‐1, thereby regulating cellular proliferation and steroidogenesis [25]. To investigate the functional role of *Igf‐1r* in folliculogenesis, we established two GCs‐specific *Igf‐1r* knockout models, a tamoxifen‐inducible knockout (*Igf‐1r^flox/flox^; Foxl2‐CreER^T2^*, termed TAM‐cKO) and a constitutive knockout (*Igf‐1r^flox/flox^; Foxl2‐Cre*, termed cKO) (Figure 6A,B). Genotype validation confirmed successful recombination in TAM‐cKO offspring (Figure 6C). IF staining confirmed GCs type‐specific *Igf‐1r* ablation in TAM‐cKO and cKO mouse ovaries. WT GCs exhibited robust nuclear IGF‐1R localization, whereas TAM‐cKO and cKO models showed near‐complete signal extinction in GCs, with residual fluorescence matching interstitial cell background levels (Figure 6D; Figure S5A). To detect the response ability of knockout mice to Gn, 3‐week‐old TAM‐cKO and cKO mice were administered with pregnant mare serum PMSG for 48 h; body weights were found to be comparable to those of the age‐matched WT group. However, a significant reduction in both ovarian weight and volume was observed in the TAM‐cKO and cKO groups (Figure 6E,F). Histological examination of ovarian tissues indicated that WT ovaries contained follicles at all stages of development, including primordial, primary, secondary, and antral follicles, thereby demonstrating normal physiological folliculogenesis. Conversely, the cKO and TAM‐cKO mice exhibited a marked decrease in the number of antral follicles relative to WT controls. Specifically, WT follicles exhibited well‐defined antral cavities surrounded by multilayered, tightly organized GCs. In contrast, TAM‐cKO and cKO follicles displayed disorganized GCs stratification and an absence of follicular cavities. cKO follicles were arrested at the secondary stage and completely lacked mature antral structures. These findings indicate that *Igf‐1r* deficiency in GCs disrupts the transition from secondary to antral follicles (Figure 6G). Similar to the findings in *Igfals* knockout mice, the conditional knockout of *Igf‐1r* in GCs also resulted in significantly downregulated expression of FSH‐responsive genes, a marked reduction in markers for antral follicles, and a significant increase in markers for preantral and atretic follicles compared to WT mice (Figure 6H; Figure S5C–P).

Given that *Igf‐1r* deficiency in GCs not only disrupted folliculogenesis but also reduced local IGF‐1 expression (Figure S5B), we next asked whether the follicular arrest resulted from impaired cellular responsiveness to IGF‐1 or was solely attributable to ligand deficiency, primary GCs were isolated from WT and TAM‐cKO mice. While exogenous IGF‐1 promoted WT GC proliferation, it failed to rescue the growth deficit in TAM cKO GCs, as measured by CCK‐8 assay and EdU incorporation assays (Figure 6I–K). These data demonstrate that specific deletion of *Igf‐1r* in GCs abrogates their responsiveness to IGF‐1, underlying the observed impairment in antral follicle development.

### 2.7 Development and Validation of an IGF‐1‐Integrated Predictive Model for Poor Ovarian Response

Serum total IGF‐1 levels are significantly lower in POR patients than NOR controls, and correlate with oocyte yield and pregnancy outcomes. Transgenic mice lacking *Igfals* or *Igf‐1r* developed POR, suggesting IGF‐1's role as a biomarker for predicting ovarian hyporesponsiveness and pregnancy chances in POR patients. The study proposes IGF‐1 as a tool for clinical strategies.

**FIGURE 7:**
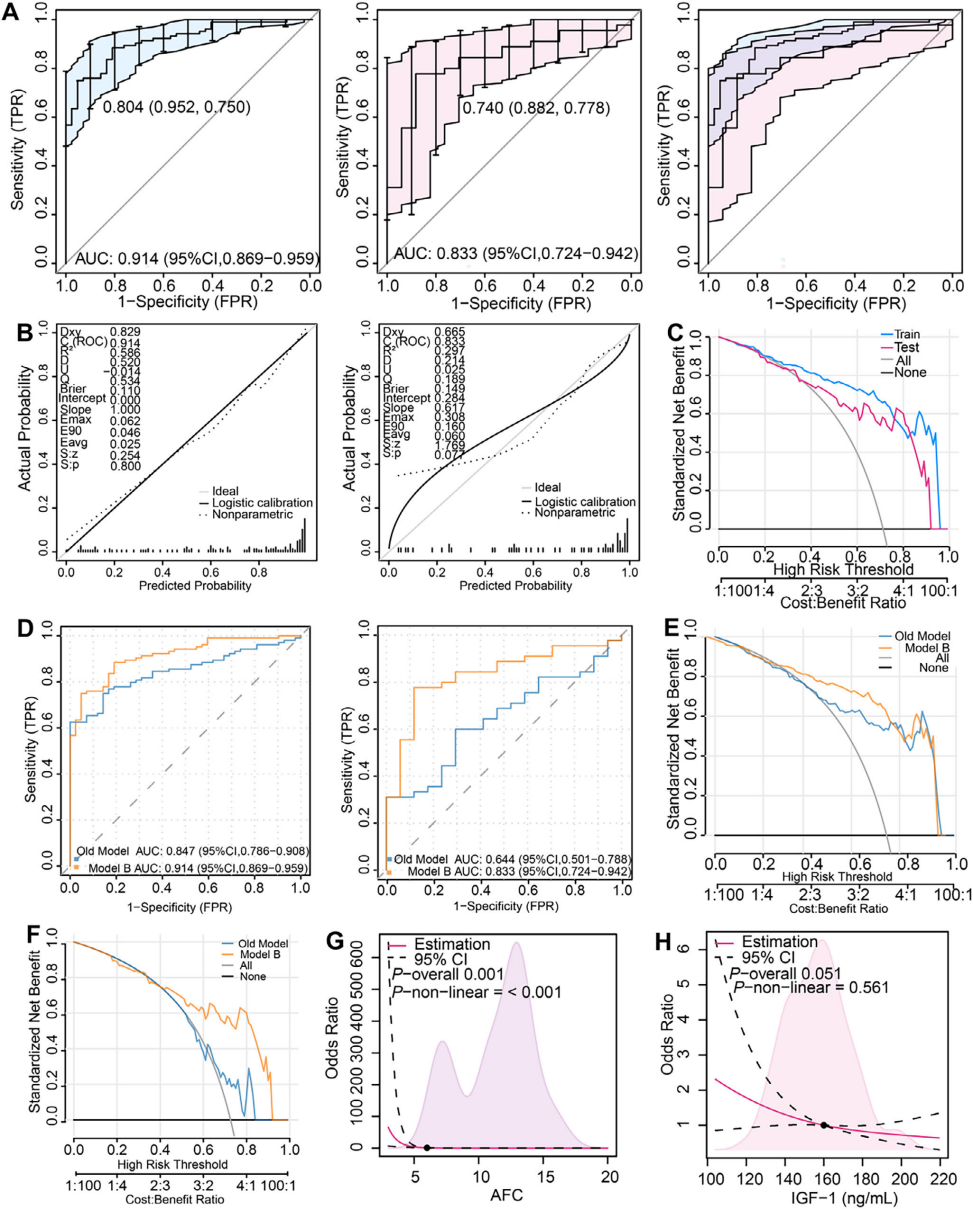
Development and validation of a clinical prediction model for POR. (A) ROC curves and AUC values for the POR prediction model in the modeling cohort (blue) and validation cohort (pink). Optimal cut‐off values for each cohort are indicated. The overlapping region of the curves is shaded purple. (B) Calibration curves for the POR clinical prediction model in the modeling cohort and validation cohort. Dashed lines represent non‐parametric calibration curves (observed vs original predicted probabilities). Solid lines represent logistic regression calibration curves (observed vs recalibrated predicted probabilities). The gray line indicates the ideal calibration line (slope = 1). (C) DCA for the POR prediction model in the modeling and validation cohorts. *X*‐axis: threshold probability. *Y*‐axis: Net benefit. Reference lines: treat all patients as non‐POR (“treat none” strategy, solid gray line); treat all patients as POR (“treat all” strategy, solid black line). Model curves: modeling cohort (solid blue line), validation cohort (solid red line). (D) ROC curves and AUC values comparing Model A (traditional predictors: AMH, age, AFC; solid blue line; modeling cohort AUC = 0.847, validation cohort AUC = 0.644) and Model B (new model; solid orange line; modeling cohort AUC = 0.914, validation cohort AUC = 0.833) in both cohorts. (E) Calibration curves for Model A (solid blue line) and Model B (solid orange line) in the modeling cohort. Solid lines depict agreement between predicted probability and observed POR incidence. Dashed line: Ideal calibration reference (y = x). Dotted lines: bootstrap confidence intervals. (F) Calibration curves for Model A (solid blue line) and Model B (solid orange line) in the validation cohort. Solid lines depict agreement between predicted probability and observed POR incidence. Dashed line: ideal calibration reference (y = x). Dotted lines: bootstrap confidence intervals. (G) Restricted cubic spline plot illustrating the nonlinear association between AFC and predicted POR probability based on Model B (shaded area: 95% confidence interval). (H) Restricted cubic spline plot illustrating the association between serum IGF‐1 level (ng/mL) and predicted POR probability based on Model B. A dashed line marks a key threshold, indicating a significant decrease in POR probability above this level (shaded area: 95% confidence interval).

Univariate logistic regression identified age, initial Gn dose, and total Gn dose as risk factors against POR, while high AMH, AFC, Gn duration, IGF‐1, and hCG day progesterone levels emerged as significant protective factors (all *p* < 0.05). These statistically significant variables were subsequently incorporated into a multivariate logistic regression model to determine independent predictors (Figure S6A,B). Lasso regression identified key factors for predicting POR (Figure S7A,B), resulting in a model based on age, Gn initiation, and total amounts, and estrogen levels on hCG day, adhering to the 10 EPV rule (Figure S7C). To use the model, determine each patient's variable values, draw lines to obtain scores, sum them, and locate the total score to find the risk of low ovarian response. The model's AUC was 0.914 for the modeling group and 0.833 for the validation group, with no significant difference between them (*p* = 0.435), indicating strong predictive performance (Figure 7A). The Precision‐Recall analysis showed an AUC of 0.9666 for the training set and 0.9323 for the validation set (Figure S8A), with low mean square errors (0.128 and 0.122) indicating high model accuracy. Calibration curves and the Hosmer–Lemeshow test confirmed good prediction accuracy, with Dxy, R^2^, and slope values showing no significant discrepancies (Figure 7B). CIC quantifies the potential clinical utility of the POR prediction model by illustrating the corresponding allocation of interventions and associated outcomes across varying high‐risk thresholds (Figure S8B). DCA revealed a net benefit of 0.05–0.95 for the model group and 0.41–0.92 for the validation group (Figure 7C). The newly developed Model B consistently exhibited superior predictive performance relative to the traditional ovarian assessment model, Model A (formerly referred to as the ‘old model’). Within the modeling cohort, Model B achieved an area under the curve (AUC) of 0.914, compared to Model A's AUC of 0.847. In the validation cohort, Model B sustained an AUC of 0.833, whereas Model A attained an AUC of 0.644. The net reclassification improvement was 0.2285 for categorical data and 0.8434 for continuous data, with an integrated discrimination improvement of 0.1811, all of which were statistically significant. Our combined model predicted a higher probability of POR compared to the traditional model (Figure 7D) and demonstrated a better fit according to the Hosmer–Lemeshow test (Figure 7E,F). Restricted cubic spline analysis indicated that patients starting COS with an AFC < 6 (Figure 7G), AMH < 1.5 ng/mL, or hCG‐day estradiol < 6800 pmol/L had a increased POR risk, with all P‐nonlinear values below 0.05 (Figure S8C,D). There was no nonlinear relationship between IGF‐1 levels and POR risk (*P*‐nonlinear = 0.561). However, a one standard deviation decrease in IGF‐1 was associated with a higher POR risk in a linear model, showing a borderline trend (*p* = 0.051). Patients with IGF‐1 levels below 160 ng/mL had a higher POR risk than those with levels at or above 160 ng/mL, indicated by an odds ratio greater than 1 (Figure 7H). The prediction model for POR established in this study exhibits superior predictive accuracy and reproducibility across multifaceted validation frameworks, demonstrating tangible potential for clinical implementation.

### 2.8 Building Predictive Pregnancy Outcome Model

Initially, a univariate logistic regression analysis was conducted to assess pregnancy outcomes (Figure S9A), identifying 12 significant factors: Age, AMH, AFC, Gn starting amount, total Gn amount, number of cycles, MII oocyte count, 2PN count, total embryos, high‐quality embryos, IGF‐1, and estrogen levels (*p* < 0.05). Multiple regression analyses refined these to five independent predictors: AMH (OR = 2.989, *p* = 0.029), MII oocyte count (OR = 3.420, *p* = 0.014), 2PN embryos count (OR = 0.299, *p* = 0.008), total embryos (OR = 3.465, *p* = 0.007), and IGF‐1 levels (OR = 1.733, *p* = 0.006) (Figure S9B) and Lasso (Figure S10A,B). The ROC and calibration curves assessed the model's discrimination and accuracy, revealing an AUC of 0.893 (95% CI: 0.834, 0.953) and accuracy of 0.835 for the model group, and an AUC of 0.891 (95% CI: 0.793, 0.988) and accuracy of 0.82 for the validation group, both with *p* < 0.001. The model showed strong discriminative ability with C‐indices of 0.891 and 0.888, respectively (Figure S10C). A nomogram was developed for predicting pregnancy outcomes. In personalized medical treatment, clinicians use a nomogram to align patient parameters like AMH levels, MII oocytes, embryos, and IGF‐1 concentrations with specific scores. By summing these scores, they estimate the probability of a successful pregnancy (Figure S10D).

**FIGURE 8:**
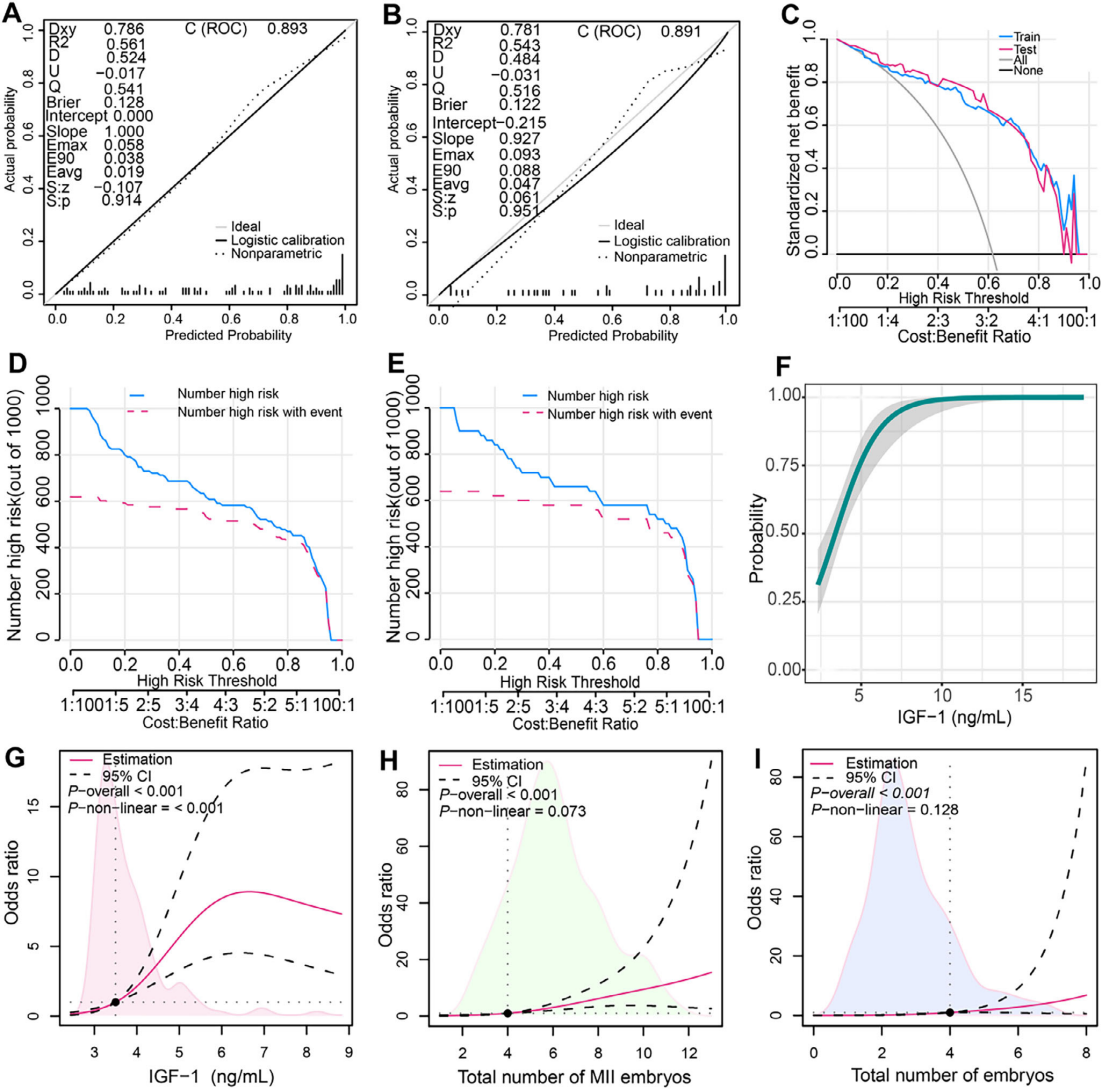
Evaluation of the pregnancy outcome prediction model for patients with POR. (A) Calibration curve for the validation cohort. *X*‐axis: predicted pregnancy probability. *Y*‐axis: actual pregnancy probability. Black dashed line: model fitting curve. Black solid line: bootstrap bias‐corrected curve. Gray solid line: ideal reference line (y = x). (B) Calibration curve for the modeling cohort. *X*‐axis: predicted pregnancy probability. *Y*‐axis: actual pregnancy probability. Black dashed line: model fitting curve. Black solid line: bootstrap bias‐corrected curve. Gray solid line: ideal reference line (y = x). (C) Clinical DCA *X*‐axis: threshold probability. *Y*‐axis: Net benefit. Gray curve: “Treat all” strategy (all patients considered pregnant). Black horizontal line: “Treat none” strategy (all patients considered not pregnant). Blue curve: modeling cohort. Red curve: validation cohort. (D) Clinical impact curve for the modeling cohort. *X*‐axis: high‐risk threshold (loss‐to‐benefit ratio indicated below). *Y*‐axis: number of high‐risk patients per 1000. Solid line: predicted number of high‐risk pregnancies. Dashed line: actual number of high‐risk pregnancies. (E) Clinical impact curve for the validation cohort. *X*‐axis: high‐risk threshold (loss‐to‐benefit ratio indicated below). *Y*‐axis: number of high‐risk patients per 1000. Solid line: predicted number of high‐risk pregnancies. Dashed line: Actual number of high‐risk pregnancies. (F) Logistic regression curve illustrating the relationship between FF IGF‐1 level and pregnancy probability (shaded area: 95% confidence interval). (G) Restricted cubic spline analysis of FF IGF‐1 levels (ng/mL) based on the model. A dashed line marks a key threshold (3.5 ng/mL), indicating a significant increase in pregnancy probability above this level (*p* < 0.001; shaded area: 95% confidence interval). (H) Restricted cubic spline analysis of MII oocyte count. A dashed line marks a key threshold (4 oocytes), indicating a significant increase in pregnancy probability above this level (*p* < 0.001; shaded area: 95% confidence interval). (I) Restricted cubic spline analysis of total embryo count. A dashed line marks a key threshold (4 embryos), indicating a significant increase in pregnancy probability above this level (*p* < 0.001; shaded area: 95% confidence interval).

The Hosmer–Lemeshow test indicated no significant difference between predicted and actual outcomes, with Dxy values of 0.786 and 0.781, and R2 values of 0.561 and 0.543. Mean square errors were 0.128 and 0.122, demonstrating good consistency and accuracy with the ideal reference curve (Figure 8A,B). Decision and impact curve analyses showed the model's highest net benefit at threshold probabilities of 0.08–0.85 and 0.2–0.95 for the model and validation groups, respectively, with a loss‐to‐benefit ratio below 1, indicating strong clinical efficacy (Figure 8C–E). A logistic curve revealed that an FF IGF‐1 level of 5 corresponded to a 75% pregnancy likelihood (Figure 8F). Restricted cubic spline analysis showed a significant non‐linear relationship between FF IGF‐1 levels and clinical pregnancy outcomes in POR patients, with rates increasing above 3.5 ng/mL, peaking at 6.5 ng/mL. No significant non‐linear associations were found between pregnancy rates and the number of MII oocytes or total embryos. However, clinical pregnancy rates improved steadily when the number of MII oocytes and embryos was 4 or more (Figure 8G–I). Collectively, these steps establish a robust and clinically applicable predictive model for pregnancy outcomes in patients with POR.

## 3 Discussion

The rising incidence and poor prognosis of POR among infertile patients undergoing human ART have emerged as a significant clinical challenge in controlled ovulation induction treatment, attracting considerable attention from researchers [26, 27, 28, 29]. Therefore, this study establishes IGF‐1 as a pivotal biomarker and mechanistic mediator underlying POR. In all participants, total serum IGF‐1 levels were higher than those in FF. However, while serum levels were only marginally reduced in POR patients compared to normal responders, the FF IGF‐1 deficiency in the POR group was substantially more pronounced. We propose that this disparity is primarily attributable to the following mechanisms: First, the vast majority of circulating IGF‐1 is bound within a large ternary complex with IGFBP‐3 and ALS. Due to the selective permeability of the blood‐follicle barrier, this complex exhibits limited diffusion into the follicular cavity. Second, FF IGF‐1 concentration represents a dynamic equilibrium between systemic endocrine input (primarily derived from hepatic synthesis) and local follicular processes involving limited synthesis and active metabolic clearance. In the pathological state of POR, compromised follicular function likely reduces the capacity for active uptake or local utilization of IGF‐1. This exacerbates the imbalance between systemic and local IGF‐1 pools, ultimately leading to a relative deficiency of this key growth factor within the follicular microenvironment. This dysregulation may constitute one mechanism underlying the diminished developmental potential of follicles in POR patients.

Crucially, FF IGF‐1 outperformed serum IGF‐1 as a biomarker of follicular competence, reflecting direct micro‐environmental dysregulation. Using GCs‐specific *Igf‐1r* knockout mice, we mechanistically linked IGF‐1R deficiency to POR pathophysiology: disrupted FSH sensitivity, suppressed estrogen synthesis, and follicular arrest at secondary stages. These insights informed the development of validated clinical nomograms integrating IGF‐1 to predict POR risk (AUC: 0.914) and pregnancy likelihood (AUC: 0.893), outperforming conventional markers.

Although the role of IGF‐1 in folliculogenesis is well‐established, its predictive value for POR has remained contested owing to methodological inconsistencies across studies. Prior reports found no FF IGF‐1 differences between pregnant/non‐pregnant POR patients [30], yet our rigorously controlled protocol, featuring single‐patient aliquots, uniform processing, limited freeze‐thaw cycles, and outcome‐blinded assays, revealed significant IGF‐1 reductions in POR cohorts. Importantly, FF IGF‐1 correlated strongly with embryo quality and pregnancy success after adjusting for age/oocyte yield, highlighting its utility as a direct follicular health indicator. Discrepancies with earlier studies likely stem from unstandardized sample handling or pooled FF analyses, which dilute IGF‐1 concentrations.

Our suite of genetically engineered mouse models, targeting distinct nodes of the IGF‐1 signaling axis, converges to delineate a unified pathophysiology that recapitulates human POR. The systemic *Igfals^−/−^* model demonstrates that a primary defect in circulating IGF‐1 bioavailability, which results from the loss of the stabilizing ternary complex, is sufficient to initiate a pathogenic cascade, depleting the hormonal support necessary for normal follicular development and leading to blunted ovarian FSH responsiveness. Conversely, granulosa cell ‐specific *Igf‐1r* knockouts establish that receptor‐level signaling is the critical conduit for IGF‐1 action within the ovary; the failure of exogenous IGF‐1 to rescue proliferation in receptor‐deficient GCs directly links the clinical observation of Gn resistance to a specific lesion in IGF‐1 signal transduction. Both models consistently result in follicular arrest beyond the secondary stage, characterized by ovarian hypoplasia and disorganized GCs, and are universally associated at the molecular level with suppressed expression of key FSH‐responsive and steroidogenic genes (e.g., *Cyp19a1, Inha*) and a transcriptional shift toward atresia (e.g., elevated *Ctgf, Cfh*). Collectively, these findings support a pathophysiological model wherein disruption of IGF‐1 signaling, regardless of whether it occurs at the level of systemic ligand bioavailability or GC‐specific receptor function, compromises the essential synergy between the IGF‐1 and FSH pathways, leading to inadequate steroidogenesis, a gene expression profile favoring developmental arrest, and ultimately the reduced yield of mature oocytes that defines the poor ovarian response to stimulation.

To bridge biology and clinical practice, we developed dual nomograms predicting POR risk and pregnancy outcomes. The POR model (integrating age, Gn dose, and IGF‐1) and pregnancy model (leveraging FF IGF‐1, AMH, MII oocytes, and embryo count) achieved superior discriminative power (AUC > 0.89) versus traditional markers (AUC <0.80). Critically, FF IGF‐1 ≥5 ng/mL predicted 75% pregnancy likelihood. These tools enable risk‐adapted ovarian stimulation (e.g., adjusted Gn dosing for high‐POR‐risk patients), individualized prognosis counseling via visual nomograms, and dynamic luteal support optimization based on pregnancy probability. This closed‐loop framework, “stratify, treat, predict” addresses a critical unmet need in ART: personalized management for POR.

This study provides a novel perspective on predicting ovarian response; however, several limitations must be acknowledged. First, its single‐center, retrospective design and relatively limited sample size entail inherent risks of selection bias and residual confounding, despite our adjustments for known confounders and internal validation via bootstrapping. The sample size, while sufficient for the primary cohort analysis, resulted in reduced statistical power for specific subgroup comparisons (e.g., age‐stratified comparisons in POR‐1/POR‐3), which were limited by small sample sizes, and results should be interpreted as exploratory. Consequently, the generalizability of our findings requires validation in prospective, multicenter, large‐scale trials involving geographically, ethnically, and clinically diverse populations. Second, the measurement of IGF‐1 was confined to the day of hCG trigger, failing to capture its dynamic fluctuations throughout the ovarian stimulation cycle (e.g., the trend from Gn initiation to hCG administration). Future studies incorporating serial measurements are needed to determine the optimal timing for prediction and to understand the kinetics of IGF‐1 during folliculogenesis. Furthermore, while the exclusion of patients with high BMI enhanced internal validity, it limits the applicability of our conclusions to non‐obese populations [31]. Expanding future research to include metabolically diverse cohorts will be crucial to elucidate broader mechanisms. Finally, our mechanistic insights remain constrained. The modest correlation between FF IGF‐1 and embryo quality implies that other determinants, such as oocyte mitochondrial function or epigenetic regulation, may exert more dominant influences. A deeper understanding necessitates subsequent research that integrates multi‐omics approaches (e.g., transcriptomics, proteomics) to systematically delineate the specific signaling pathways and regulatory networks governed by IGF‐1 in folliculogenesis and embryogenesis.

## 4 Conclusion

In conclusion, this work establishes IGF‐1 deficiency as a biomarker and mechanistic driver of POR. By transforming this biological insight into validated clinical tools, we provide a pathway for personalized management of ovarian insufficiency, positioning IGF‐1 not only as a diagnostic sentinel but as a promising therapeutic axis in reproductive medicine.

## 5 Materials and Methods

### 5.1 Animals

*Igf‐1r^flox/flox^* mice were kindly gifted by Professor Haibing Wang (Xiamen University, Fujian, China). *Foxl2‐CreER^T2^* mice were generously provided by Professor Hua Zhang (China Agricultural University, Beijing, China). *Foxl2‐Cre* mice were generously provided by Professor Fengchao Wang (the Transgenic Animal Center of the National Institute of Biological Sciences, Beijing, China). *Igfals*‐KO mice were generously provided by GemPharmatech Co., Ltd. (Nanjing, Jiangsu, China), generated via the CRISPR/Cas9 genome‐editing technology. All animals were maintained under specific pathogen‐free conditions with controlled environmental parameters: 12/12 h light/dark cycle, 50%–70% humidity, and ambient temperature of 22 ± 2°C. For all experiments, comparisons were made between experimental mice and their littermate controls. For conditional knockout induction, *Igf‐1r^flox/flox^* and *Igf‐1r^flox/flox^; Foxl2‐CreER^T2^* female mice received intraperitoneal injections of tamoxifen (T5648, Merck, USA) at 20 mg/kg body weight every other day from postnatal day 14, totaling three administrations. Tamoxifen was prepared as a 100 mg/mL stock solution in 95% ethanol (v/v), then diluted to 20 mg/mL with corn oil (8001‐30‐7, Klamar, China) and stored at ‐20°C protected from light. The first 12 h after birth were considered as 0 day postpartum (dpp). Three administrations were delivered on 14, 16, and 18 dpp. Control mice received equivalent volumes of corn oil vehicle. Ovaries were collected 72 h after the final injection (21 dpp). Mice were intraperitoneally injected with 5 IU of PMSG (Sansheng Biological Technology, China) 48 h before sample collection. All animal experiments were conducted in strict accordance with the ARRIVE guidelines. Animal protocols were approved by the Institutional Animal Care Committee of Guizhou Medical University (Ethics No. 2402754).

### 5.2 Study Design and Patient Population

This retrospective case‐control study included patients undergoing their first IVF‐ET or intracytoplasmic sperm injection (ICSI) cycle at Reproductive Medicine Center of the Affiliated Hospital of Guizhou Medical University (January 2023 – June 2024). Inclusion criteria comprised women aged 20–45 years with regular cycles undergoing IVF/ICSI solely for tubal factors. Exclusion criteria were: BMI >25 kg/m^2^ [32]; ICSI for male factor or PGT; PCOS/suspected high responders; hypertension, cardiovascular, cerebrovascular, systemic diseases; hydrosalpinx; oral contraceptive use or smoking within 3 months prior to hormone testing; prior ovarian surgery, ovarian cysts, or unilateral oophorectomy; recurrent miscarriage; incomplete data (e.g., missing AMH); uterine factors (e.g, adhesions, submucosal fibroids, hyperplasia, malformations, polyps, or endometrial thickness ≤7 mm); or concurrent endometriosis, primary ovarian insufficiency (POI), premature ovarian failure (POF), diabetes, or infections. POR were stratified per 2016 POSEIDON criteria: [5] POR‐1 (35 year, AFC≥ 5, AMH≥ 1.2 ng/mL, unexpected poor response); POR‐2 (≥ 35 year, AFC≥ 5, AMH≥ 1.2 ng/mL, unexpected poor response); POR‐3 (35 year, AFC< 5, AMH< 1.2 ng/mL, oocytes retrieved > 1 and ≤ 9); POR‐4 (≥ 35 year, AFC< 5, AMH< 1.2 ng/mL, oocytes retrieved >1 and ≤ 9). The normal ovarian responder (NOR) group comprised women < 35 years with ≥ 10 but < 15 oocytes retrieved and AMH >1.2 ng/mL. The information about the patient was listed in Tables S1 and S2. The study was approved by the Guizhou Medical University Ethics Committee (Approval No. 2024–57), registered at ChiCTR (ChiCTR250009555), and obtained written informed consent from all participants.

### 5.3 Defining IVF‐ET Pregnancy Outcomes

Pregnancy outcomes following IVF‐ET were rigorously defined according to established clinical criteria. Serum estrogen and progesterone levels were assessed on the day of hCG administration, and endometrial thickness was measured on the day of embryo transfer. Biochemical Pregnancy: Defined by a serum β‐hCG level of ≥ 50 U/L measured 14 days after embryo transfer, in the absence of subsequent ultrasound confirmation of a clinical pregnancy. Clinical Pregnancy: Confirmed by the transvaginal ultrasonographic visualization of one or more gestational sacs (including ectopic pregnancies) with a detectable fetal heartbeat, typically performed at 5–7 weeks of gestation. Early Pregnancy Loss: This composite endpoint encompassed all pregnancies that did not progress beyond the first trimester, including biochemical pregnancies, ectopic pregnancies, and miscarriages (i.e., the loss of a clinical pregnancy before 12 weeks of gestation). Clinical Pregnancy Rate: Was calculated as the number of cycles resulting in a clinical pregnancy per 100 embryo transfer cycles.

### 5.4 Construction of Clinical Prediction Models

We developed two distinct clinical prediction models using harmonized methodology: POR Prediction Model: Utilizing serum total IGF‐1 levels and clinical parameters from 208 IVF cycles (7:3 stratified split), with comparable baseline characteristics between groups (Table S3). 71.2% of the modeling cohort and 72.6% of the validation cohort exhibited POR. Pregnancy outcome model: Incorporating FF IGF‐1 from 165 cycles, demonstrating balanced demographics (Table S4) with pregnancy rates of 51.2% (modeling) and 56.8% (validation).

Both models employed binary logistic regression with feature selection through univariate screening (*p* < 0.05) followed by stepwise multivariate regression (AIC‐optimized). LASSO regularization (R v4.1‐4; λ‐optimized) and nomogram construction (standardized coefficients) addressed overfitting. Validation protocols included hold‐out testing, ten‐fold cross‐validation (repeated), leave‐one‐out validation, and bootstrap resampling (*n* = 1000). Model performance was rigorously assessed via calibration curves, DCA (clinical utility), and Clinical Impact Curves (CIC). Partial figures were generated using the LogisticAppOutput software.

### 5.5 Ovarian Stimulation Protocol

On menstrual cycle days 2–3, hormone levels (FSH, LH, E_2_, P, T, PRL, AMH) were measured, and an ultrasound confirmed antral follicles ≤ 6.0 mm. A personalized Gn protocol was designed based on BMI, age, AFC, previous response, and FSH levels, using either a GnRH agonist (Decapeptyl, IPSEN PHARMA, France) or antagonist (Cetrotide, Merck Serono, Germany). Stimulation began on days 2–3 after pituitary down‐regulation or progestogen withdrawal, with hormone tests and ultrasound. The initial Gn dose (Gonal‐f, Merck Serono, Germany) was based on AFC, AMH, age, and BMI. Monitoring started 4–5 days after stimulation, using transvaginal ultrasound to track endometrial thickness and follicle growth, and measuring serum E_2_, P, and LH to adjust rFSH dosage. Recombinant hCG (Ovitrelle, 250 µg, Merck Serono, Switzerland) was used for triggering when follicles matured (≥1 at 18 mm, ≥2 at 17 mm, or ≥3 at 16 mm). Oocyte retrieval occurred 36 h later, guided by ultrasound, with serum IGF‐1 measured simultaneously. Luteal phase support with intramuscular progesterone began the day after. Fresh embryo transfer was done on day 3 or 5 post‐retrieval, based on patient age, cycle history, and clinical needs.

### 5.6 Collection of FF

FF was collected under transvaginal ultrasound guidance during oocyte retrieval. The probe was positioned to visualize the endometrium, bilateral ovaries, and mature follicles, with optimization of scanning parameters (magnification, gain, and puncture guide overlay). A calibrated puncture trajectory minimized the distance between the vaginal fornix and target ovary, confirming ovarian position, follicle count, and diameter (cross‐validated against ovulation records). A single dominant follicle (≥ 18 mm diameter) was punctured along the guideline, and the first clear FF aliquot was aspirated. Samples were immediately transferred to the embryology laboratory and processed under individualized protocols: FF from each patient was collected into a dedicated sterile polypropylene tube, adhering to a strict single‐patient‐per‐tube principle. All processing steps, from follicular fluid aspiration to final aliquot storage, were completed within a 30 min timeframe to preserve sample integrity. Briefly, the fluid was poured into a 60 mm culture dish for identification of cumulus‐oocyte complexes (COCs) under a stereomicroscope. COCs were collected using a 37°C‐preheated Pasteur pipette. The remaining fluid was subsequently filtered through a 300 µm cell strainer, transferred to pre‐chilled tubes on ice, and centrifuged at 1500 × g for 10 min at 4°C. The resulting supernatant was aliquoted (500 µL) into RNase‐free tubes and immediately stored at ‐80°C.

### 5.7 Isolation of Human Primary GCs

The GCs layer at the interface was meticulously extracted and subjected to a single wash with 2 mL of phosphate‐buffered saline (PBS). In instances where red blood cells were detected within the cell precipitate, an additional wash using 2 mL of red blood cell lysis buffer (R1010, Solarbio, China) was conducted. Subsequently, the samples underwent two further washes with PBS (3 mL, 500 × g, 5 min). All steps were performed promptly after FF collection, with the entire processing completed within 30 min. The resulting pellets were aliquoted into pre‐chilled, RNase‐free tubes for storage at ‐80°C. In the analysis of pregnancy outcomes within this study, samples were only thawed and analyzed after a clinical pregnancy was confirmed in patients. Patients were subsequently categorized into a pregnancy group or a non‐pregnancy group based on the final outcome. To ensure the accuracy of causal inferences, cases with incomplete follow‐up or ambiguous outcomes were excluded.

### 5.8 Generation of Stable KGN Cell Lines with Modulated IGF‐1/IGF‐1R Expression

Stable KGN cell lines with altered expression of IGF‐1 or IGF‐1R were established via lentiviral transduction. Four lines were generated: *IGF‐1*‐overexpressing (*IGF‐1*‐OE), IGF‐1R‐overexpressing (*IGF‐1R*‐OE), IGF‐1‐knockdown (*IGF‐1*‐KD), and IGF‐1R‐knockdown (*IGF‐1R*‐KD) cells. For overexpression, full‐length human *IGF‐1* or *IGF‐1R* cDNA was cloned into a lentiviral expression vector. For knockdown, short hairpin RNA (shRNA) sequences targeting *IGF‐1* or *IGF‐1R* were designed and inserted into a lentiviral shRNA vector. The constructed plasmids, along with packaging plasmids (psPAX2 and pMD2.G), were co‐transfected into HEK‐293T cells using Lipofectamin 3000 (L3000001, Invitrogen, USA) to produce lentiviral particles. Virus‐containing supernatant was collected 48–72 h post‐transfection, filtered, and concentrated. KGN cells were transduced with the respective lentiviral preparations at a multiplicity of infection of 10–20 in the presence of 8 µg/mL polybrene. After 48 h, stable pools were selected with 15 µm puromycin (ST551, Beyotime, China) for 3–4 days. Successful generation of each cell line was confirmed by Western blot analysis of whole‐cell lysates using antibodies against IGF‐1 or IGF‐1R, with β‐actin serving as a loading control.

### 5.9 Isolation of Primary Granulosa Cells from Mouse Ovaries

Ovarian granulosa cells were isolated using a mechanical method. Following euthanasia of 3‐week‐old female mice, ovaries were aseptically dissected and placed in ice‐cold phosphate‐buffered saline (PBS). Under a stereomicroscope, the ovaries were meticulously cleaned by removing the surrounding oviduct and adipose tissue. The cleaned ovaries were then transferred to a sterile culture dish and repeatedly punctured with a 27‐gauge needle to release the granulosa cells. The liberated cells were washed from the dish with PBS, passed through a cell strainer to remove debris, and centrifuged at 300 × g for 5 min. The resulting pellet was collected for subsequent experiments.

### 5.10 HE Staining

Ovaries were fixed at 4°C in 4% paraformaldehyde for 24 h, dehydrated through a graded ethanol series (70%‐100%), cleared in xylene, and embedded in paraffin. Serial sections (5 µm thickness) were cut using a Leica RM2235 microtome and mounted on charged slides. For HE staining, sections were deparaffinized in xylene, rehydrated through ethanol gradients, and stained with Harris hematoxylin (G1080, Solarbio, Beijing, China) for 3 min, followed by eosin Y (318906, Sigma–Aldrich, China) for 1 min. Digital images were acquired using an Olympus BX63 epifluorescence microscope (Tokyo, Japan). Follicle staging was performed according to established criteria [33].

### 5.11 Ovarian Tissue IF

Ovarian sections were deparaffinized with xylene and rehydrated through a descending ethanol series. Heat‐induced epitope retrieval was performed in 10 mm sodium citrate buffer (pH 6.0) using a pressurized decloaking chamber at 95°C for 15 min. After blocking with 5% BSA in PBS (0.01 m, pH 7.4) for 60 min at 25°C, sections were incubated with primary antibodies (Table S5) at 4°C for 16 h, followed by species‐matched secondary antibodies conjugated with HRP (1:500; Abcam) at 37°C for 60 min. Three rigorous PBS‐T washes (0.1% Tween‐20, 15 min each) were performed between steps. Fluorescence signals were captured using an Olympus FV3000 confocal system and quantified via ImageJ (NIH) with background subtraction using the rolling ball radius algorithm (50 pixels).

### 5.12 Enzyme‐Linked Immunosorbent Assay

Concentrations of IGF‐1 in human FF and mouse serum, along with serum levels of E2 and IGF in mice, were measured using ELISA. All assays were performed using commercially available ELISA kits. Human FF samples were centrifuged to remove cellular debris and subsequently filtered through a 0.22 µm membrane prior to analysis. Each sample was assayed in duplicate. The following commercial kits were employed: human FF IGF‐1 was quantified using the Human IGF‐1 ELISA Kit (EK1131‐98T, Multi Sciences, China, sensitivity: 71.18 pg/mL). For mouse serum analyses, the following kits were used: Jianglai Mouse E2 ELISA Kit (JL11790‐48T, Jianglai, China; detection range: 0.78–50 pg/mL); FanKeWei Mouse IGF‐1 ELISA Kit (F9411‐A‐48T, FanKeWei, China; detection range: 0.7–25 µg/L); and FanKeWei Mouse free IGF ELISA Kit (F0686‐MA‐48T, FanKeWei, China; detection range: 0.5–26 µg/L). The lower limit of quantification and both intra‐ and inter‐assay coefficients of variation for each kit fell within the ranges specified by the manufacturer. Data obtained from experimental groups were normalized to those of the control group.

### 5.13 Western Blotting analysis

The protein concentration in the GCs, determined by the Bradford method, ranged from 60 to 90 mg/mL. Samples were prepared at a concentration of 1 µg/µL, with a loading volume of 10 µL, resulting in a total protein content of 10 µg. Equal amounts of total protein from each group were subjected to SDS‐PAGE electrophoresis and subsequently transferred onto methanol‐activated PVDF membranes. The membranes were blocked with 5% skim milk and incubated overnight at 4°C with primary antibodies. The primary antibodies were listed in Table S5. Following the washing of the primary antibody, the PVDF membranes were incubated with the corresponding secondary antibody at room temperature for a duration of 2 h. Subsequent to washing the secondary antibody, the sample was exposed to an ECL chemiluminescence solution, and image acquisition was conducted using a chemiluminescence imaging instrument (Tian5200, Tanon, China). The integrated optical density of the protein bands was quantified using ImageJ analysis software.

### 5.14 Cell IF

Human primary GCs were cultured in F‐12 medium for 12 h, washed twice with PBS, and fixed in 4% paraformaldehyde (4°C, 4 h). Cells were permeabilized with 0.1% Triton X‐100 (P0096‐100, Beyotime, China) in PBS for 30 min, then incubated with primary antibodies at 4°C for 12 h. After two PBS washes (100 rpm, 15 min total), secondary antibodies were applied at 37°C for 1 h, followed by three PBS washes (15 min each). The primary antibodies were listed in Table S5. Nuclei were stained with DAPI (C1005, Beyotime, China) for 5 min and washed twice with PBS. Images were captured using an Olympus confocal microscope with consistent intensity/contrast settings (≥ 50 cells/group, random fields), and fluorescence intensity was quantified with ImageJ.

### 5.15 qPCR

Total RNA was isolated from ovarian tissues using TRIzol Reagent (15596018CN; Thermo Fisher Scientific, Waltham, MA, USA). Following extraction, this RNA underwent reverse transcription into complementary DNA (cDNA) with HiFi first Strand cDNA Synthesis SuperMix (11141ES60; YEASEN, China), strictly following the manufacturer's protocol. qPCR amplification was then conducted using HieffUNICON Universal Blue qPCR SYBR Green Master Mix (11184ES60, YEASEN) on an Applied Biosystems 7300 Real‐Time PCR System (Life Technologies, Carlsbad, CA, USA). Primer sequences employed in this study are detailed in Table S6.

### 5.16 Cell Viability Assay (CCK‐8)

Cell viability was assessed by CCK‐8 assay. Cells were trypsinized, seeded in 96‐well plates at 5 × 10^3^ cells/well, and allowed to adhere for 24 h. Outer wells contained PBS to minimize evaporation. Blank (medium only), control (cells only), and treatment (cells treated with recombinant lGF1 protein) groups were included, with at least six replicates per condition. After treatment, 10 µL CCK‐8 (k1018, APEXBIO, USA) solution was added to each well. Plates were incubated at 37°C until the OD450 of control wells reached approximately 1.0. Absorbance was measured at 450 nm. For the cell viability assay, KGN cells were cultured with 100 ng/mL recombinant FSH (Sigma–Aldrich) for 24 h to mimic the in vivo exposure of follicular granulosa cells to gonadotropin stimulation.

### 5.17 EdU Assay for Cell Proliferation

Cells were seeded on coverslips in six‐well plates at 1 × 10^4^ cells/well and cultured to ∼60% confluence. Click‐iT EdU Imaging Kit (CA1170, Solarbio Biotechnology, China) (10 µm) was added for 3 h, followed by fixation with 4% PFA for 15 min and permeabilization with 0.3% Triton X‐100 for 10 min. The Click‐iT reaction mix was applied for 30 min in the dark. After washing, nuclei were stained with DAPI (1 µg/mL, 10 min). Coverslips were mounted with anti‐fade medium and imaged within 24 h.

### 5.18 Statistical Analysis

All experiments were conducted with three independent replicates, and results were consistent across repetitions. Statistical analyses were performed using GraphPad Prism (version 8.0) and R (version 4.3.2). The normality of distribution for all continuous variables was assessed using the Shapiro–Wilk test, and homogeneity of variances was evaluated with Levene's test. Based on these assessments, continuous data are presented as mean ± standard deviation if normally distributed, or as median (interquartile range) if non‐normally distributed. For comparisons between two groups of normally distributed data, an independent samples t‐test was used. For comparisons among more than two groups of normally distributed data with equal variances, one‐way analysis of variance (ANOVA) was applied, followed by an appropriate post‐hoc test (e.g., Tukey's) when significant. If the data violated the assumptions of normality or homogeneity of variances, non‐parametric tests were employed: the Mann–Whitney U test for two‐group comparisons and the Kruskal–Wallis test for multiple groups, with Dunn's test for post‐hoc analysis. Categorical data are summarized as frequencies and percentages, and group differences were analyzed using the Chi‐square test or Fisher's exact test, as appropriate. The primary outcomes of this study are count data, including the number of oocytes retrieved, MII oocytes, 2PN zygotes, and high‐quality embryos. A Score test for overdispersion indicated significant deviation from the Poisson assumption (O‐value > 1.96, *p* < 0.05). Therefore, negative binomial regression models were used to analyze these outcomes, with results expressed as incidence rate ratios (IRRs) alongside their 95% confidence intervals. A two‐sided *p*‐value of less than 0.05 was considered statistically significant. The entire analytical process was conducted in a blinded manner, with data preprocessing and formal statistical analysis performed independently by different investigators to minimize bias.

## Author Contributions

Z.H. performed the conceptualization, formal analysis, and methodology, provided resources, and wrote the original draft. Y.Y. carried out the investigation and validation and contributed to review and editing. G.H., J.L., C.Y., A.L., and T.C. contributed to conceptualization and methodology. J.T. contributed to conceptualization. S.Z. and T.Z. contributed to conceptualization, formal analysis, and methodology, supervised the project, and contributed to review and editing.

## Funding

This work was supported by Guizhou Provincial Science and Technology Projects (QKHJC [2024] Youth 271), Pilot Program‐China Reproductive Health Public Welfare Fund Project (NO. SZ202408), the Scientific Research Project from the Health Commission of Guizhou Province (gzwkj2025‐095), the National Natural Science Foundation of China (82260291), the Affiliated Hospital of Guizhou Medical University National Natural Science Foundation Cultivation Project (gyfynsfc[2024]‐13), Innovation and Entrepreneurship Training Program for College Students in Guizhou Province (2024106600509).

## Ethics Approval and Consent to Participate

All experimental procedures were approved by the Ethics Committee of the Affiliated Hospital of Guizhou Medical University and the Animal Research Committee of Guizhou Medical University.

## Conflicts of Interest

The authors declare no conflict of interest.

## Supporting information




**Supporting File**: advs73968‐sup‐0001‐SuppMat.docx.

## Data Availability

The data that support the findings of this study are available from the corresponding author upon reasonable request.
