# Supplementary material for: IGF‐1 Deficiency Serves as an Integrated Biomarker Pathogenic Driver and Predictor in Poor Ovarian Response
Source: Adv Sci (Weinh). 2026 Jan 29;13(18):e14483. doi: 10.1002/advs.202514483 (PMC13042885; doi:10.1002/advs.202514483)
Supplement: Supplementary file 1 — Supporting File: advs73968‐sup‐0001‐SuppMat.docx. [file ADVS-13-e14483-s001.docx]

**IGF-1 Deficiency Serves as an Integrated Biomarker Pathogenic Driver and Predictor in Poor Ovarian Response**

**Supplemental figure legends**

**
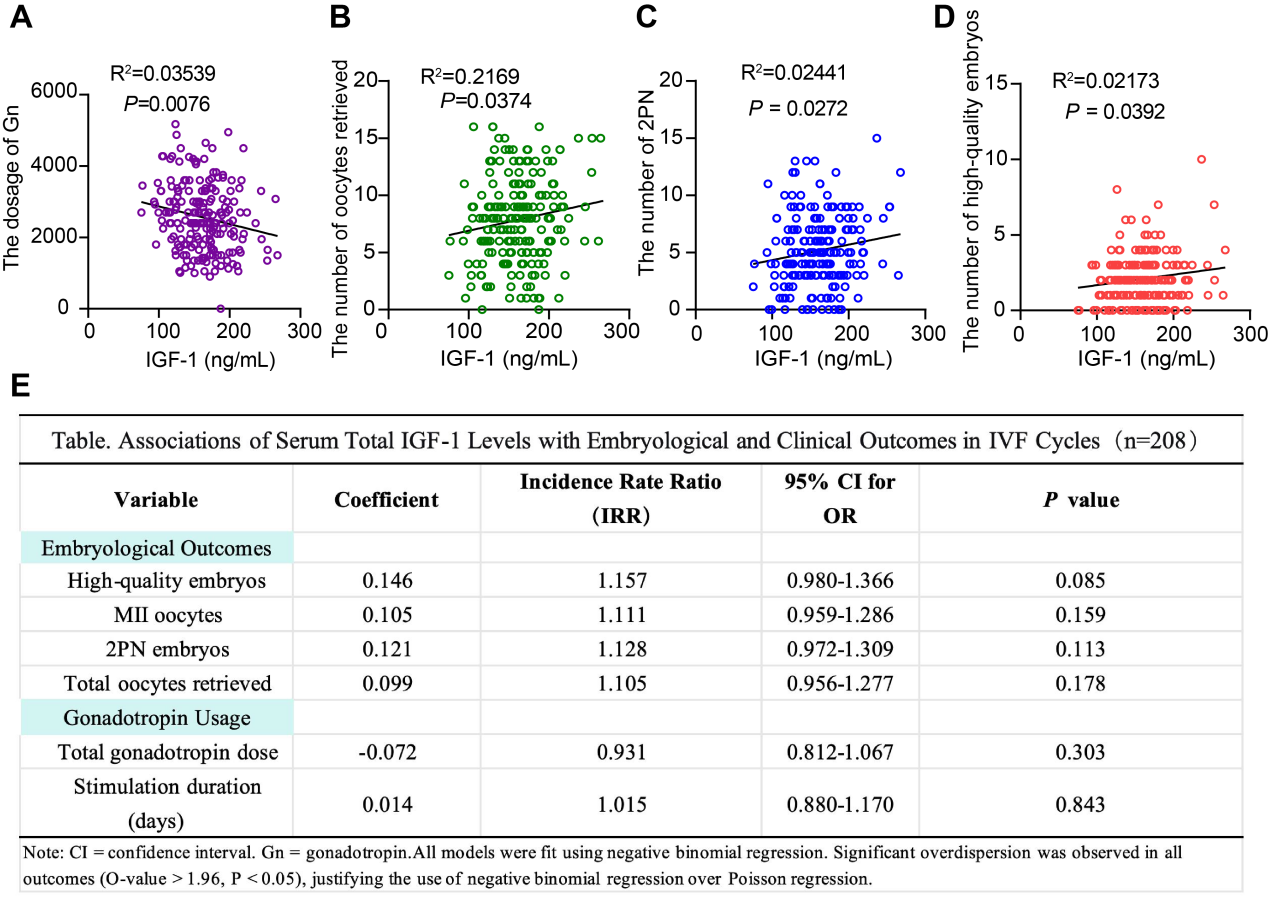
**

**Figure S1. Regression analyses of serum total IGF‑1 levels and key IVF laboratory parameters.**

**(A-D)** Linear regression analysis. Serum total IGF‑1 levels showed p‑values <0.05 but low R^2^ values (range: 0.02173‑0.2169) against (A) total gonadotropin dose, (B) oocyte yield, (C) 2PN count, and (D) number of high‑quality embryos, indicating weak linear correlations. **(E)** Negative binomial regression analysis. Serum total IGF‑1 level showed no significant association with any parameter (all *P* > 0.05). For high‑quality embryo count, the association approached but did not reach significance (*P* = 0.085; IRR = 1.157).

**
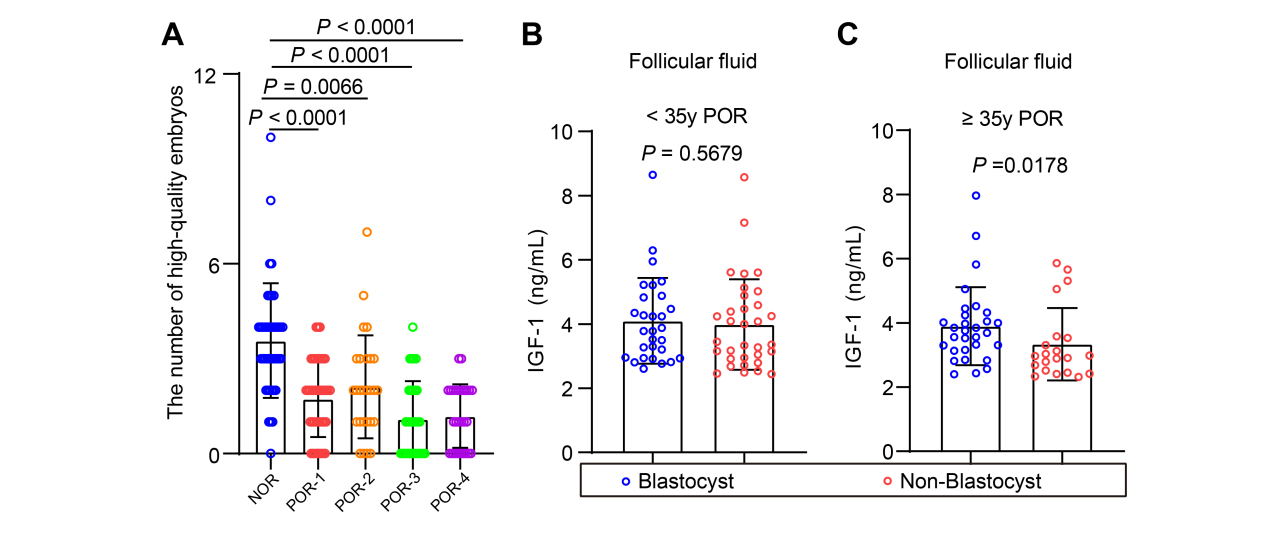
**

**Figure S2. Relationship between IGF-1 levels, ovarian response, and embryo quality.**

1. Number of high-grade embryos in NOR (n=42) versus POR subgroups (POR-1, n=41; POR-2, n=27; POR-3, n=31; POR-4, n=27). **(B-C)** FF IGF-1 levels in POR patients <35 years (B) and ≥35 years (C) with or without blastocyst formation. Data are presented as mean ± SEM. Statistical significance was determined by the Kruskal-Wallis test with Dunnett’s post hoc test for multiple comparisons in (A), and by the Mann-Whitney U test for two-group comparisons in (B) and (C). Exact *P*-values are shown on the graphs.


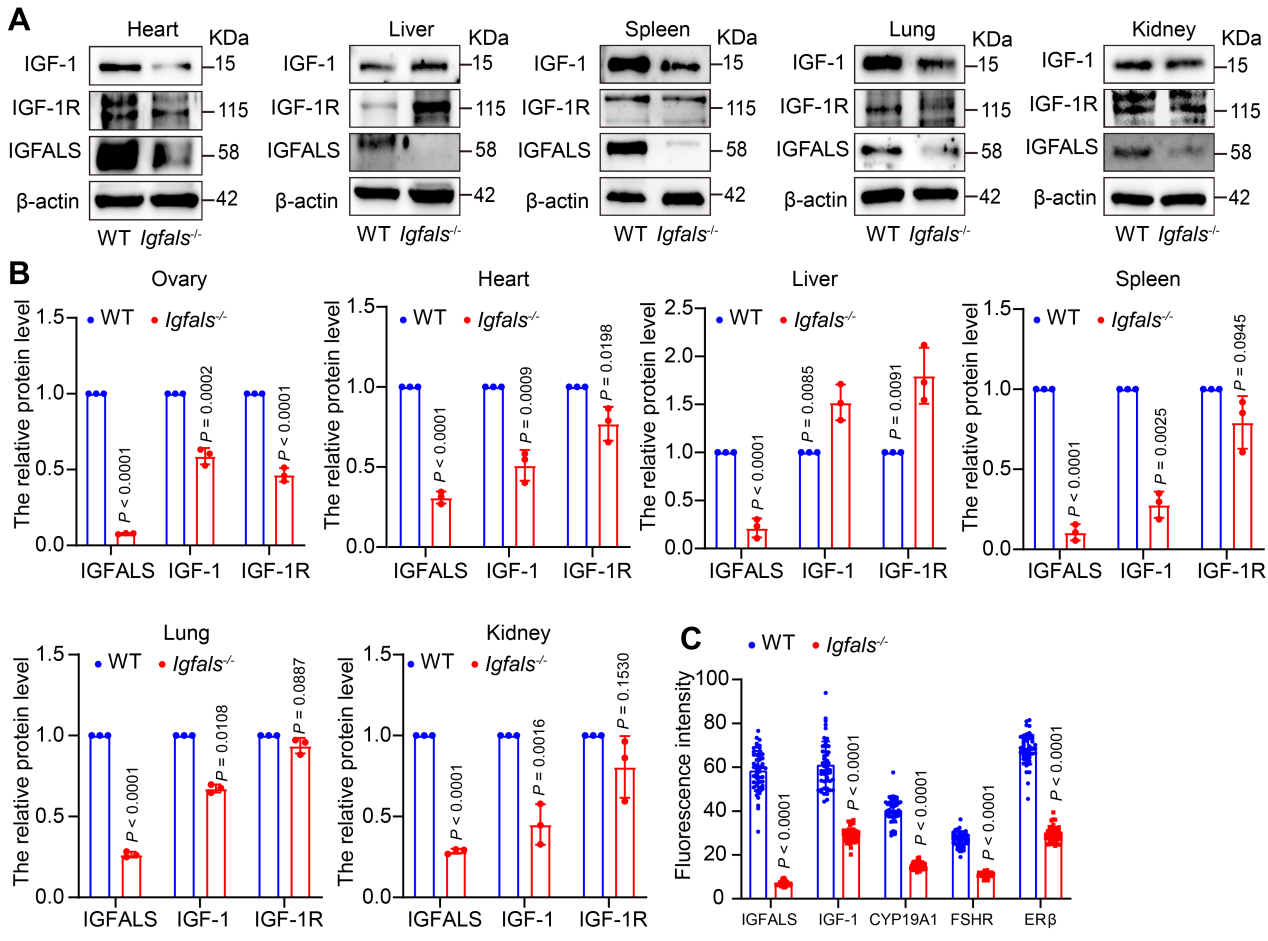


**Figure S3. Systemic and tissue-specific effects of *Igfals* knockout on the IGF-1 pathway.**

**(A)** Representative Western blot analysis of IGFALS, IGF-1, and IGF-1R protein levels in heart, liver, spleen, lung, and kidney from WT and *Igfals^-/-^* mice. β-actin serves as a loading control. IGFALS is ablated across all tested tissues, with tissue-specific alterations in IGF-1 and IGF-1R. **(B)** Quantitative analysis of Western blot signals from the multi-organ corresponds to Figure 4B and Figure S3A, highlighting the tissue-specific response to systemic IGFALS loss. n=3. **(C**) Quantification of mean fluorescence intensity for IGFALS, IGF-1, CYP19A1, FSHR, and ERβ from random fields (n=50 cells/group) confirms a significant reduction in these proteins. For quantitative data in (B) and (C), values are presented as mean ± SEM. Statistical significance was determined by two-tailed unpaired Student's t-test, and exact *P*-values are indicated on the graphs.


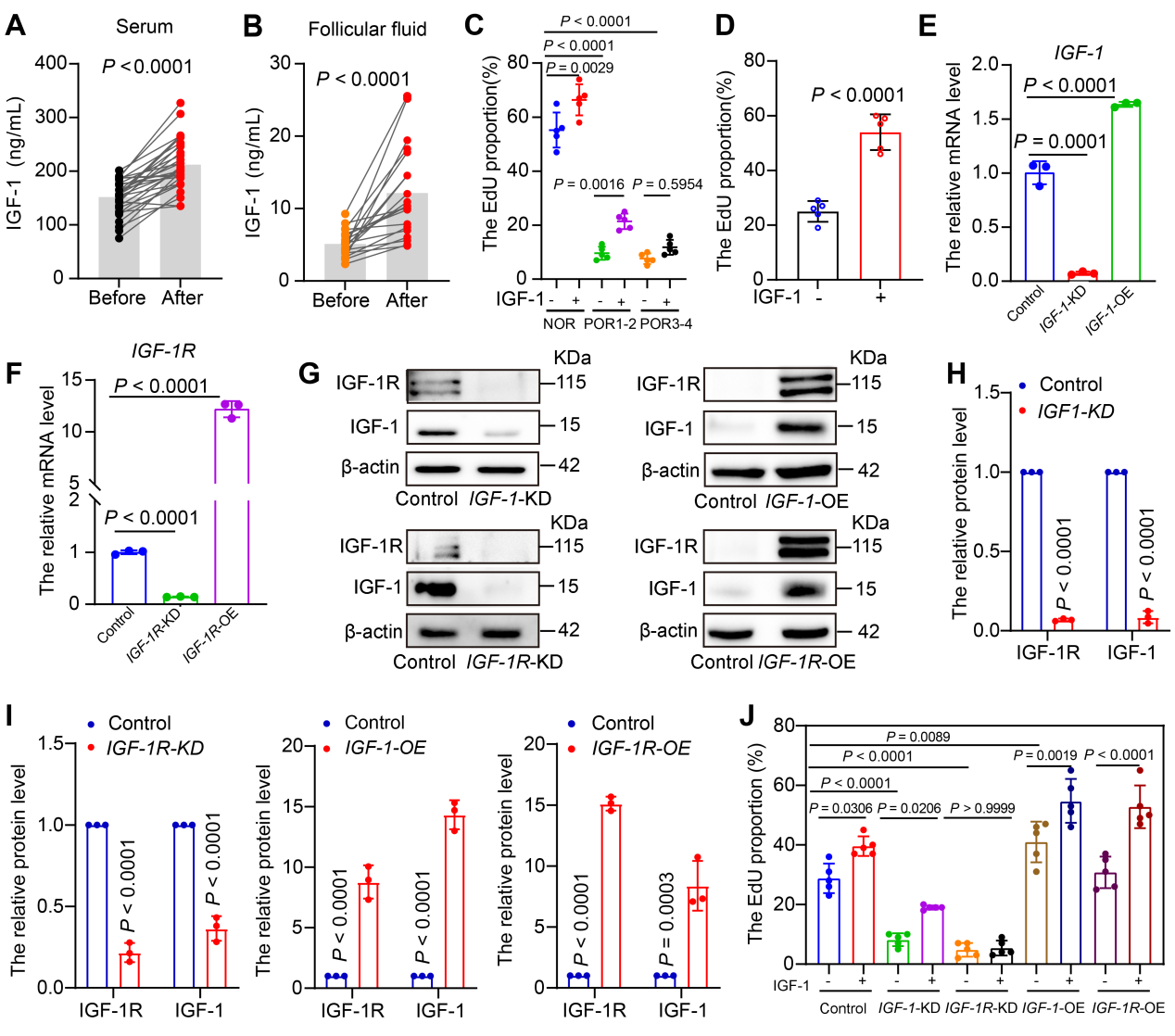


**Figure S4 Validation of engineered KGN cell models with modulated IGF-1 signaling.**

**(A-B)** Paired analysis of serum (A) and FF (B) IGF-1 levels in POR patients before and after somatropin treatment (n=20 and n=30, respectively). **(C)** Proliferation of NOR- and POR-derived GCs with or without recombinant lGF1 protein (60 ng/mL), measured by EdU incorporation (n=3). **(D)** Proliferation of mouse primary GCs treated with recombinant lGF1 protein (60 ng/mL) versus control (n=3). **(E-F)** qPCR validation of IGF-1 (I) and IGF-1R (J) mRNA levels in engineered KGN cells (control, KD, OE; n=3). **(G-I)** Immunoblot analysis and quantification of IGF-1 and IGF-1R protein expression in *IGF-1-KD, IGF-1R-KD, IGF-1-OE and IGF-1R-OE* KGN cells (n=3). **(J)** Quantification of EdU-positive cells in control, *IGF-1-KD, IGF-1R-KD, IGF-1-OE and IGF-1R-OE* KGN lines with or without recombinant lGF1 protein (60 ng/mL; n=3). Data are presented as mean ± SEM unless noted. Statistical tests used: Kruskal–Wallis with Dunnett’s post hoc (A); Mann-Whitney U test (B, C); one-way ANOVA or Kruskal–Wallis with post hoc pairwise comparisons (D, M); paired t-test (E, F); two-way ANOVA with Tukey’s test (G) or Šidák’s test (N); unpaired t-test (H, I, L). Exact *P*-values are indicated on graphs.

**
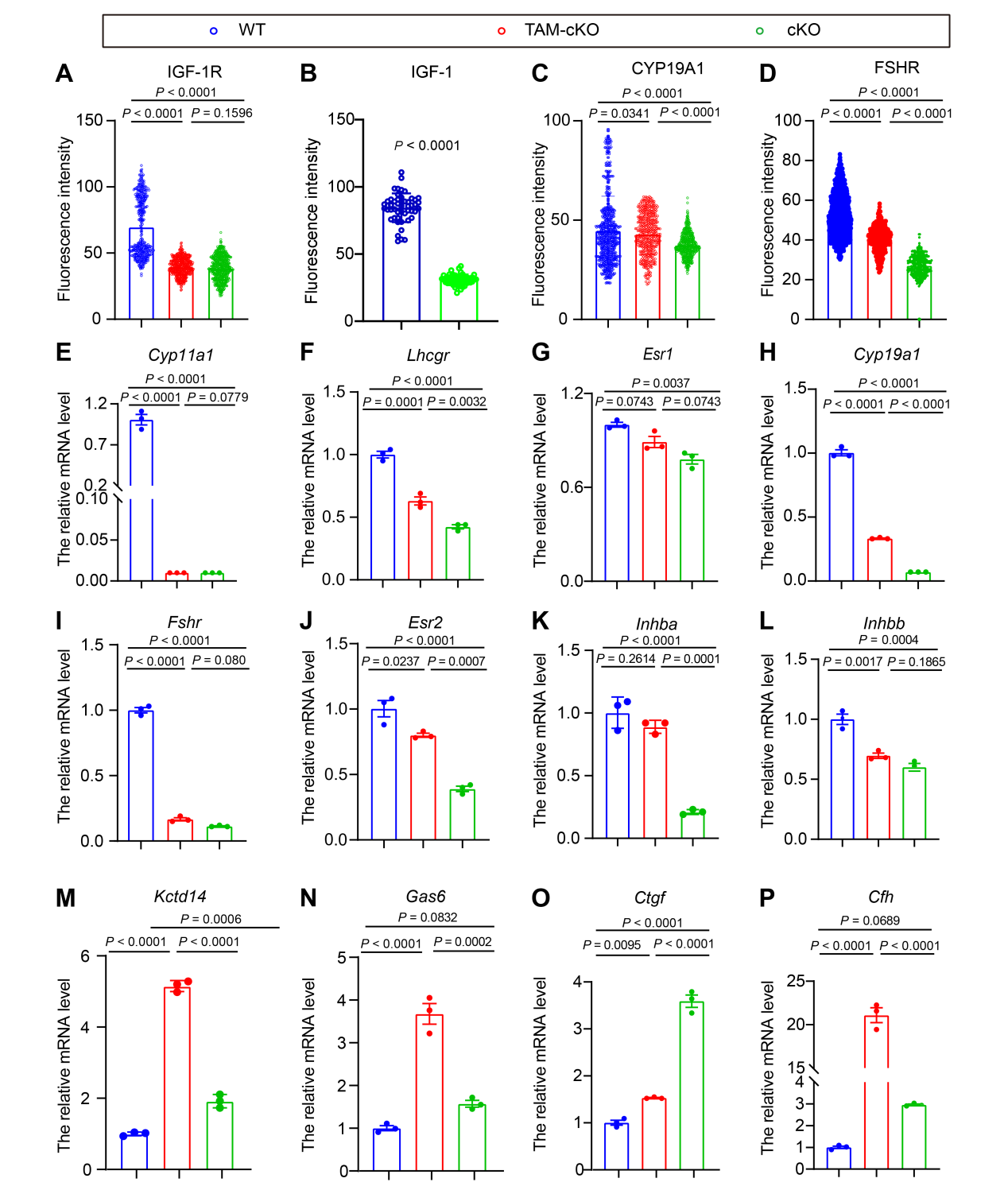
**

**Figure S5. Loss of functional identity in *Igf-1r* deficient GCs leads to follicular arrest.**

**(A)** IGF-1R IF in ovarian tissues of WT, TAM-cKO, and cKO mice, demonstrating knockout efficiency. **(B-D)** Expression levels of IGF-1 (B) CYP19A1 (C), and (D) FSHR were assessed under the same conditions. For all panels, fluorescence intensity was quantified from five random fields per biological replicate (n = 3 mice), with >200 cells analyzed per condition. Data (mean fluorescence intensity) are presented as mean ± SEM and were compared by one-way ANOVA with Tukey's post hoc test. **(E-J)** qPCR analysis of FSH-responsive gene mRNA expression in the three genotypes. (E) *Cyp11a1*. (F) *Lhcgr*. (G) *Esr1.* (H) *Cyp19a1*. (I) *Fshr.*(J) *Esr2* mRNA levels were normalized to β-actin and are shown as mean ± SEM (n = 3 biological replicates). **(K-P)** Expression analysis of follicular development markers: antral follicle markers (*Inhba*, *Inhbb*), preantral follicle markers (*Kctd14*, *Gas6*) and atretic follicle markers (*Ctgf*, *Cfh*). For all panels, statistical comparisons were performed using one-way ANOVA with Tukey's post hoc test.

**
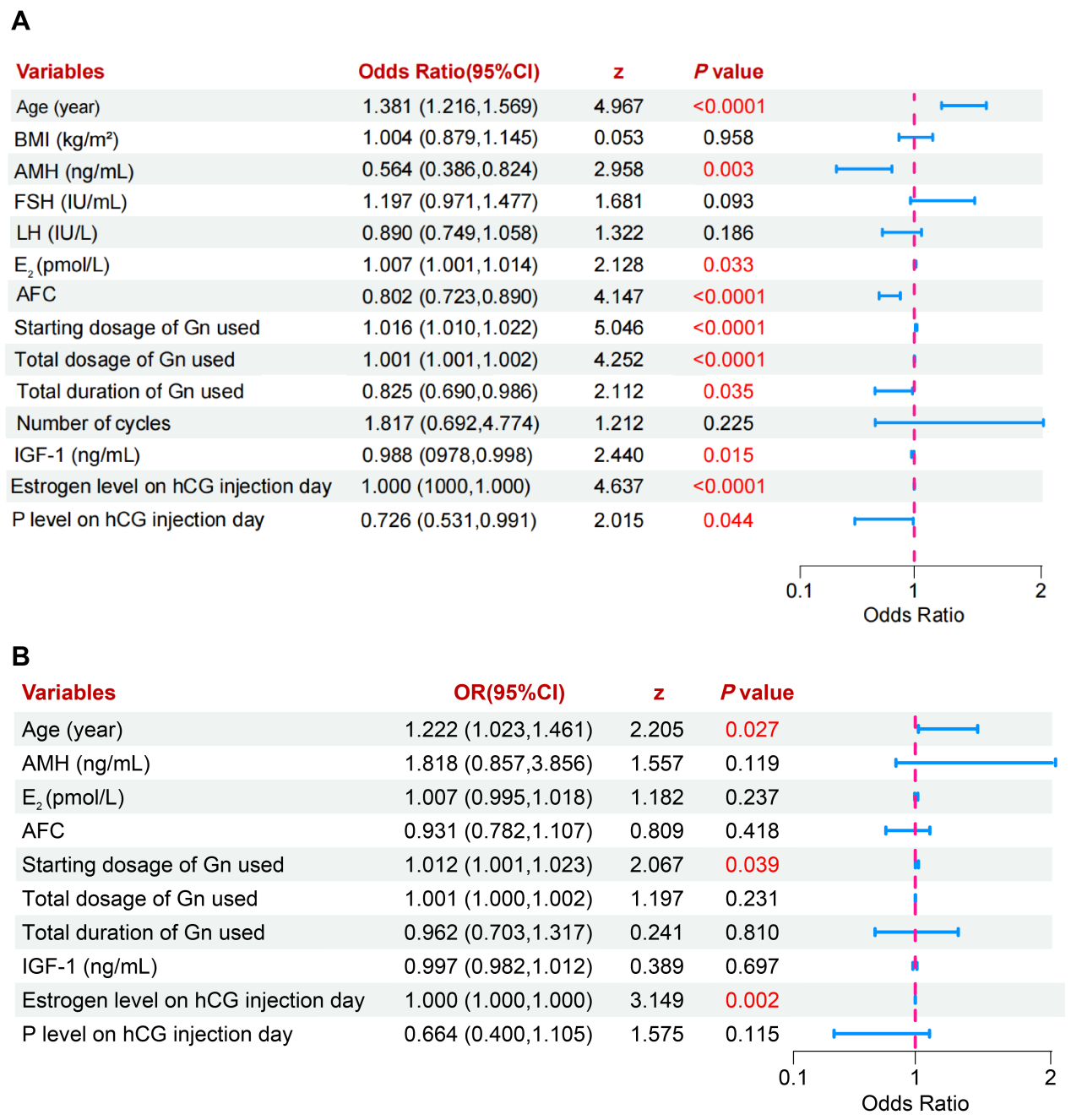
**

**Figure S6. Development process and key determinants of the POR clinical prediction model**.

**(A)** Candidate variables significantly associated with POR (*P*<0.05) were identified using univariable logistic regression analysis. **(B)** Forest plot derived from multivariable logistic regression analysis, depicting the final independent predictors incorporated into the model along with their effect sizes (Odds Ratios, OR) and 95% confidence intervals (95% CI). The dashed line indicates the reference at OR=1.

**
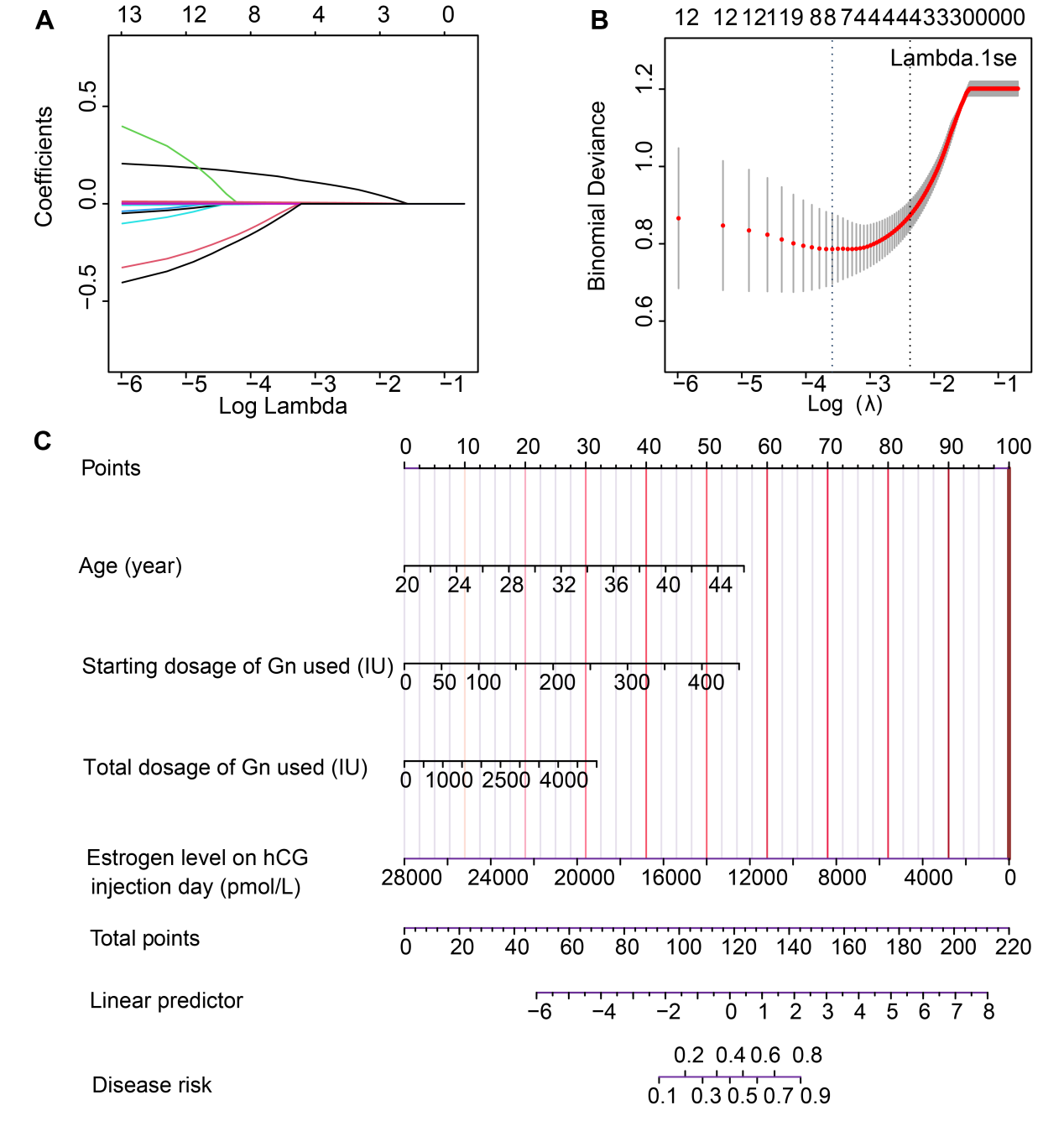
**

**Figure S7. Construction of the POR prediction nomogram.**

1. Plot of ten-fold cross-validation for identifying the optimal predictors: The x-axis represents the natural logarithm of the optimal tuning parameter (ln (λ)); the y-axis represents the binomial deviance. The final predictors were selected based on the λ value corresponding to the minimum deviance plus one standard error (λ 1se). **(B)** Lasso regression coefficient path plot for POR determinants: The x-axis represents the logarithm of the optimal tuning parameter (log λ); the y-axis represents the standardized regression coefficients (Coefficients). The vertical line in the plot indicates the position of λ 1se. **(C)** Nomogram for the POR prediction model: Patient age, starting Gn dose, total Gn dose, and serum estradiol level on the day of hCG administration were identified as independent predictors of POR following initial screening by univariable analysis, variable selection via Lasso regression, and validation by multivariable regression. Based on specific values of these four independent predictors (Patient age, serum E_2_ on hCG day, starting Gn dose, total Gn dose), an individual score (Points) is calculated for each factor. The scores for all factors are summed to yield a total score (Total Points). The probability of an individual developing POR (Probability) is predicted on the lower axis according to the total score.

**
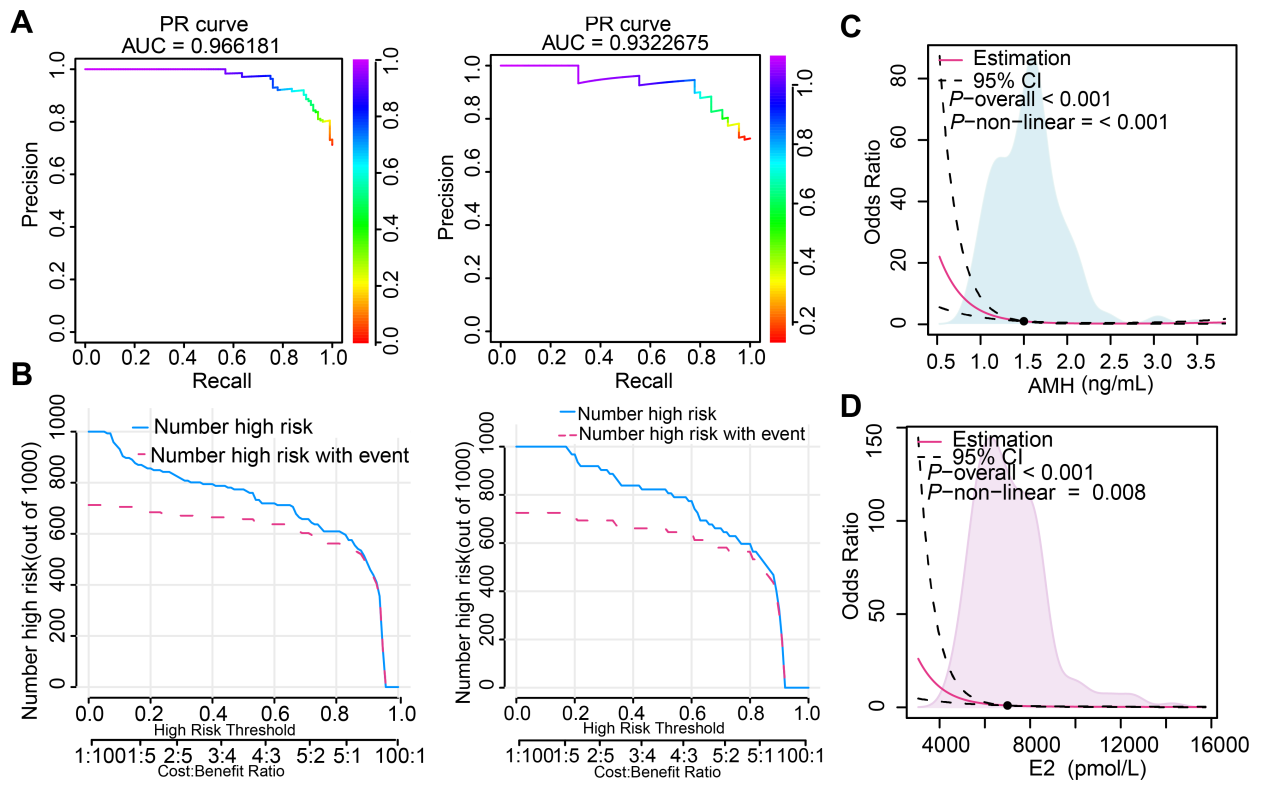
**

**Figure S8. Evaluation of the prediction model.**

**(A)** Precision-Recall (PR) curve of the POR prediction model in the training set and validation set. **(B)** Clinical impact curve for the training set and validation set: The x-axis represents the high-risk threshold, with the number below indicating the loss-to-benefit ratio; the y-axis represents the number of risk patients per 1000. Solid line: Predicted number of high-risk pregnancies; Dashed line: Actual number of high-risk pregnancies. **(C)** Model-based restricted cubic spline analysis of Anti-Müllerian Hormone (AMH, ng/mL): Dashed line marks the critical threshold (1.5 ng/mL). POR significantly decreased when AMH > 1.5 ng/mL (*P* < 0.001). **(D)** Restricted cubic spline analysis of estrogen level on hCG injection day: Dashed line marks the critical threshold (7000 pmol/L). POR significantly decreased when estrogen on hCG day > 7000 pmol/L (*P* < 0.001).

**
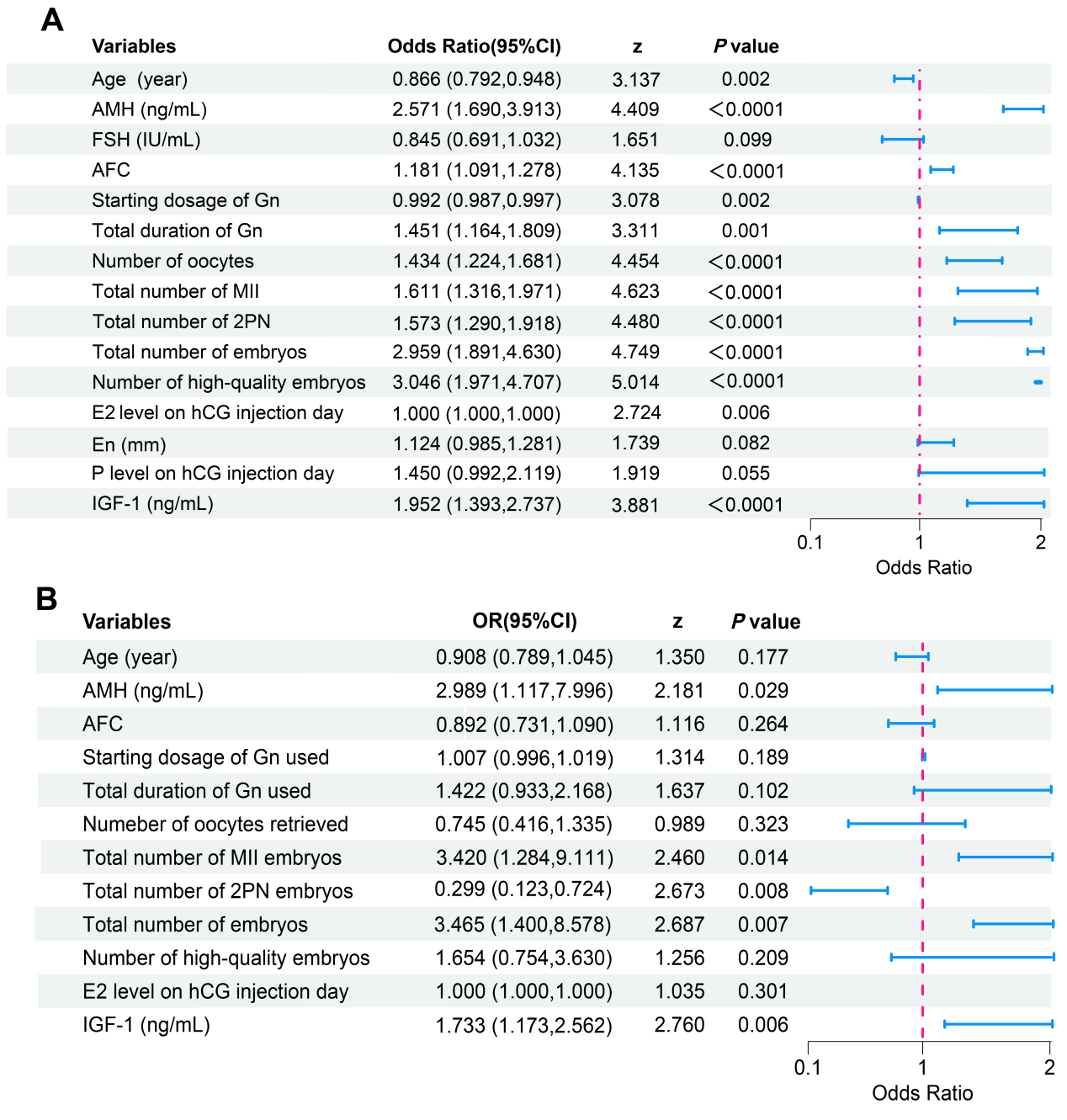
**

**Figure S9. Development process and independent determinants of the POR clinical pregnancy outcome prediction model.**

1. Screening of candidate predictors significantly associated with clinical pregnancy outcome based on univariate logistic regression analysis (*P* < 0.05). **(B)** Forest plot from multivariable logistic regression analysis demonstrating the final independent determinants incorporated into the model, including their effect sizes [Odds Ratios (OR) and 95% Confidence Intervals (95% CI)]. The dashed line indicates the reference line at OR=1.

**
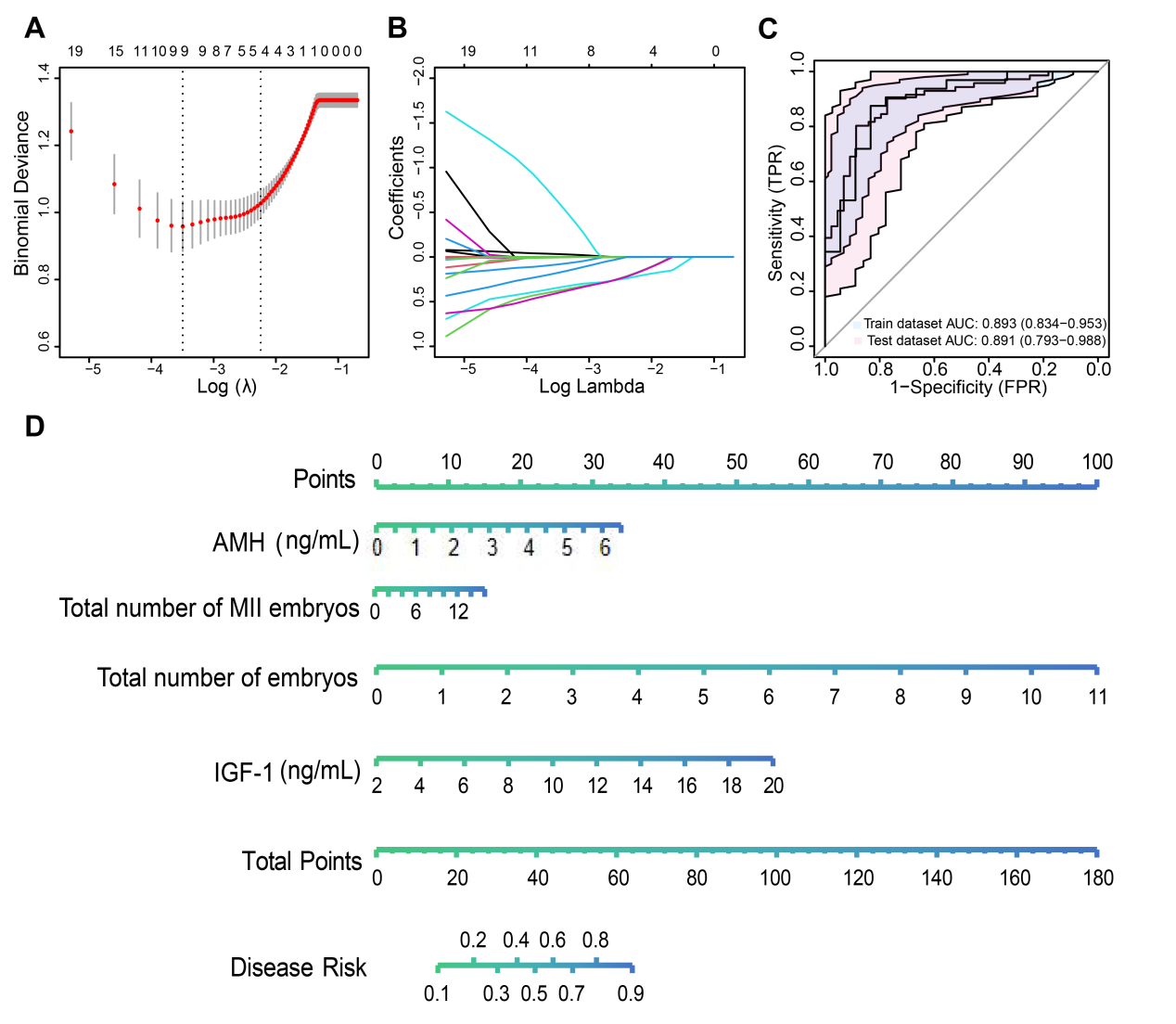
**

**Figure S10. Development, validation, and clinical translation of the POR clinical prediction model.**

1. Screening curve based on ten-fold cross-validation for λ value selection [X-axis: ln(λ); Y-axis: Binomial deviance; λmin corresponds to the minimum deviance]. **(B)** LASSO regression coefficient path plot [X-axis: log(λ); Y-axis: Standardized coefficients; Vertical line: Position of λmin from (A)]. **(C)** ROC curve [X-axis: 1 - Specificity (False positive rate); Y-axis: Sensitivity; AUC value annotated; Modeling cohort (purple) and Validation cohort (pink)]. **(D)** Clinical nomogram: Following initial screening by univariate analysis, variable selection via LASSO regression, and validation by multivariable analysis, four independent predictors were identified (AMH [Anti-Müllerian Hormone], number of MII oocytes, total number of embryos, FF IGF-1). An individual score (Points) is calculated based on the value of each predictor. These scores are summed to yield a total score (Total Points), which maps to the probability of an individual developing POR on the bottom probability axis.

**Table S1. Baseline Characteristics of Patients in Serum Sample.**

**
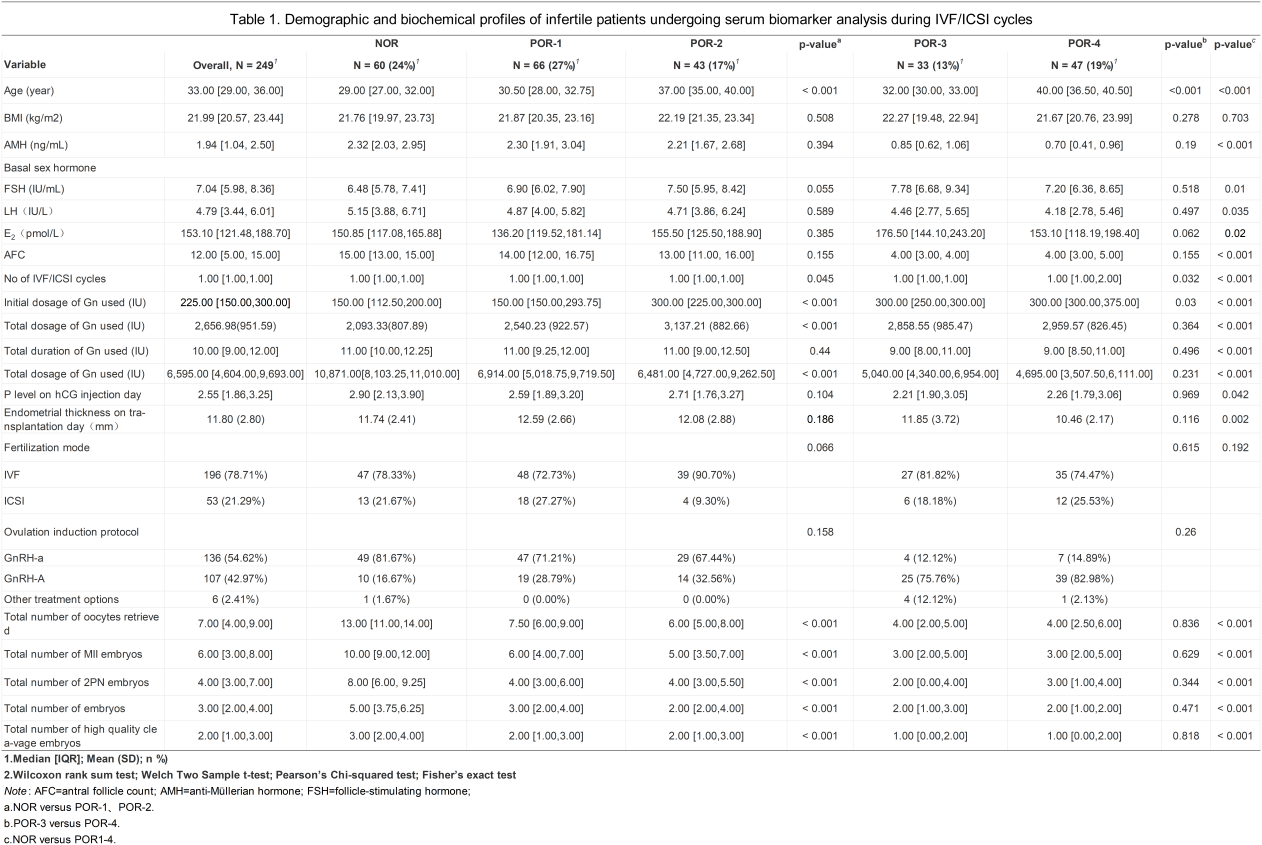
**

**Table S2. Clinical Indicators of Patients in Five Groups After Controlled Ovulation Induction.**

**
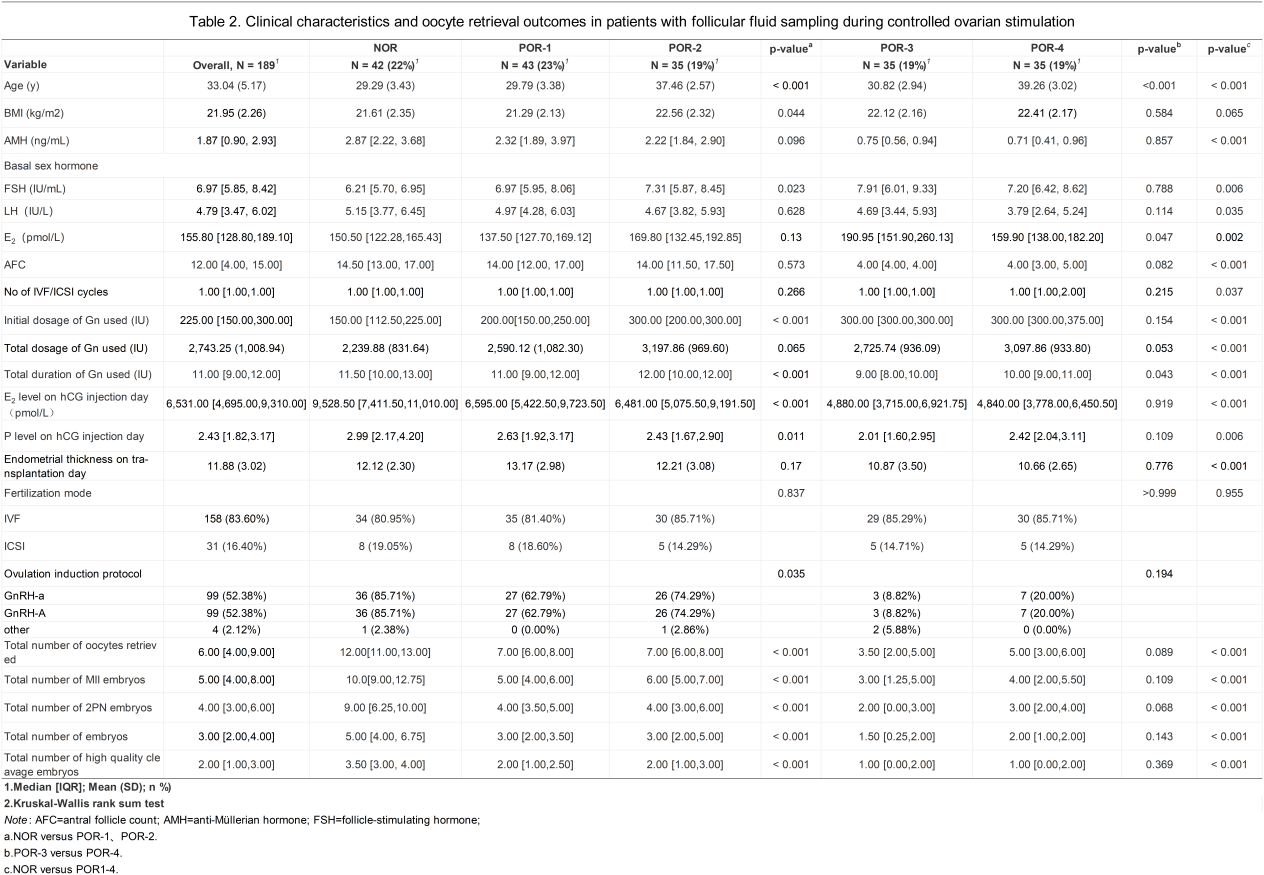
**

**Table S3. Associations of FF IGF-1 Levels with Key Embryological and Clinical Parameters in IVF (n=168).**

**
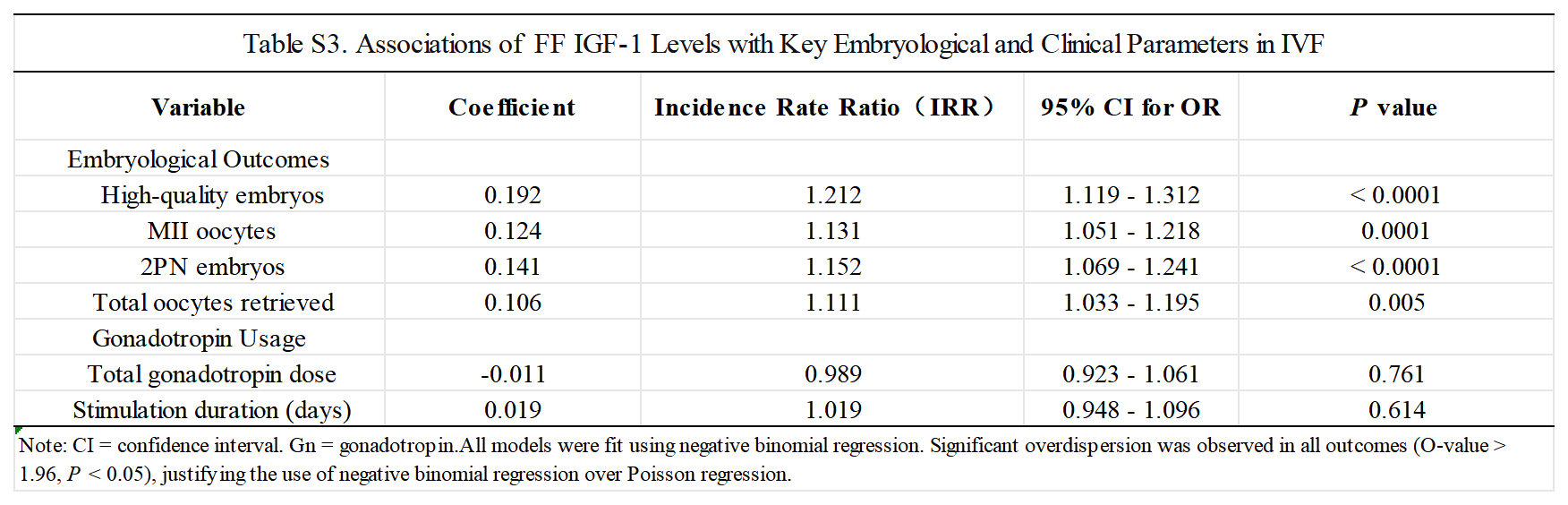
**

**Table S4. Comparison of Baseline Data and Clinical Characteristics of Infertility Patients Between the Two Groups.**

**
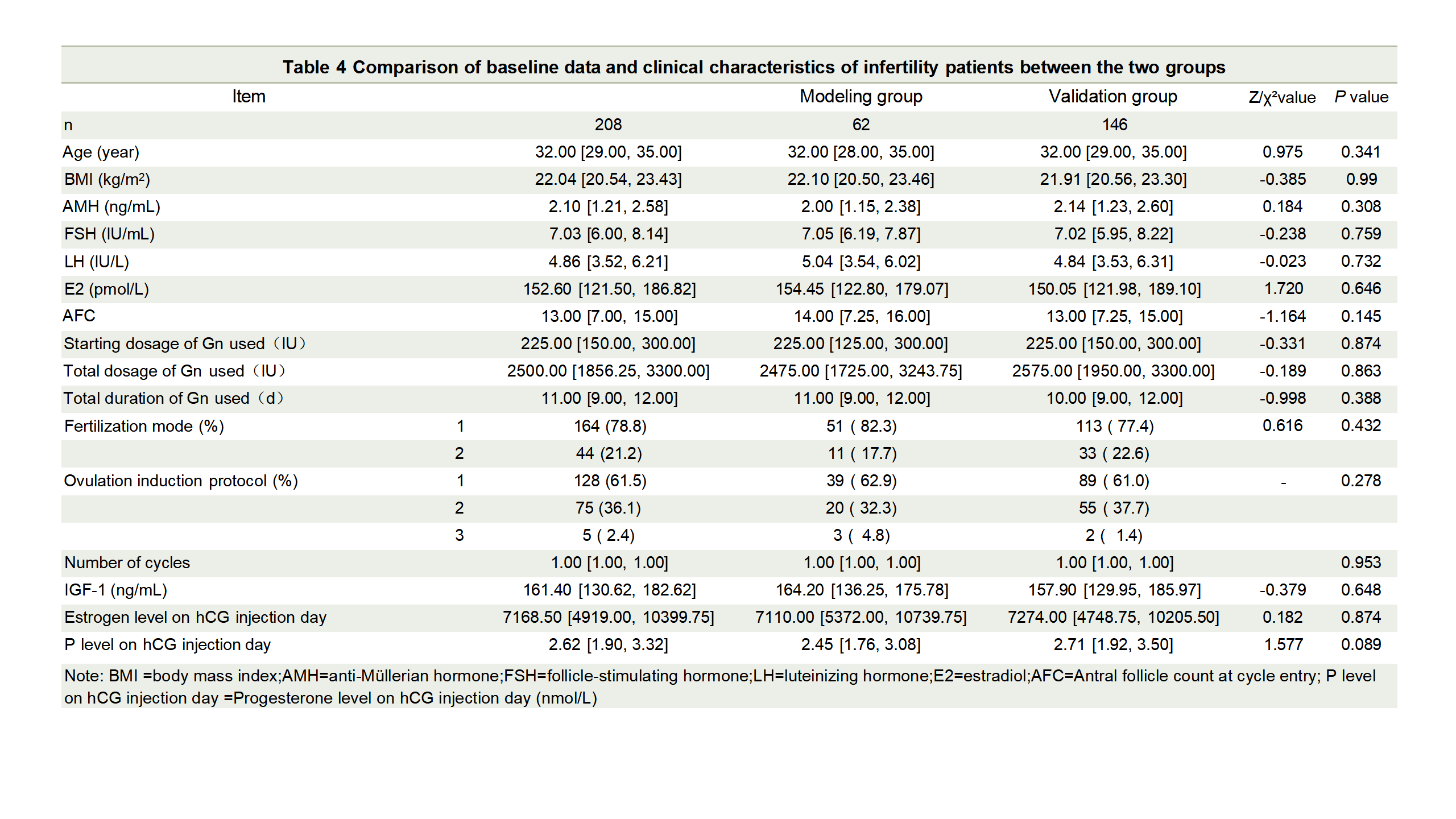
**

**Table S5 Comparison of Baseline Data, Preconception Disease Status and Clinical Characteristics Between Pregnant Group and Non-pregnant group.**


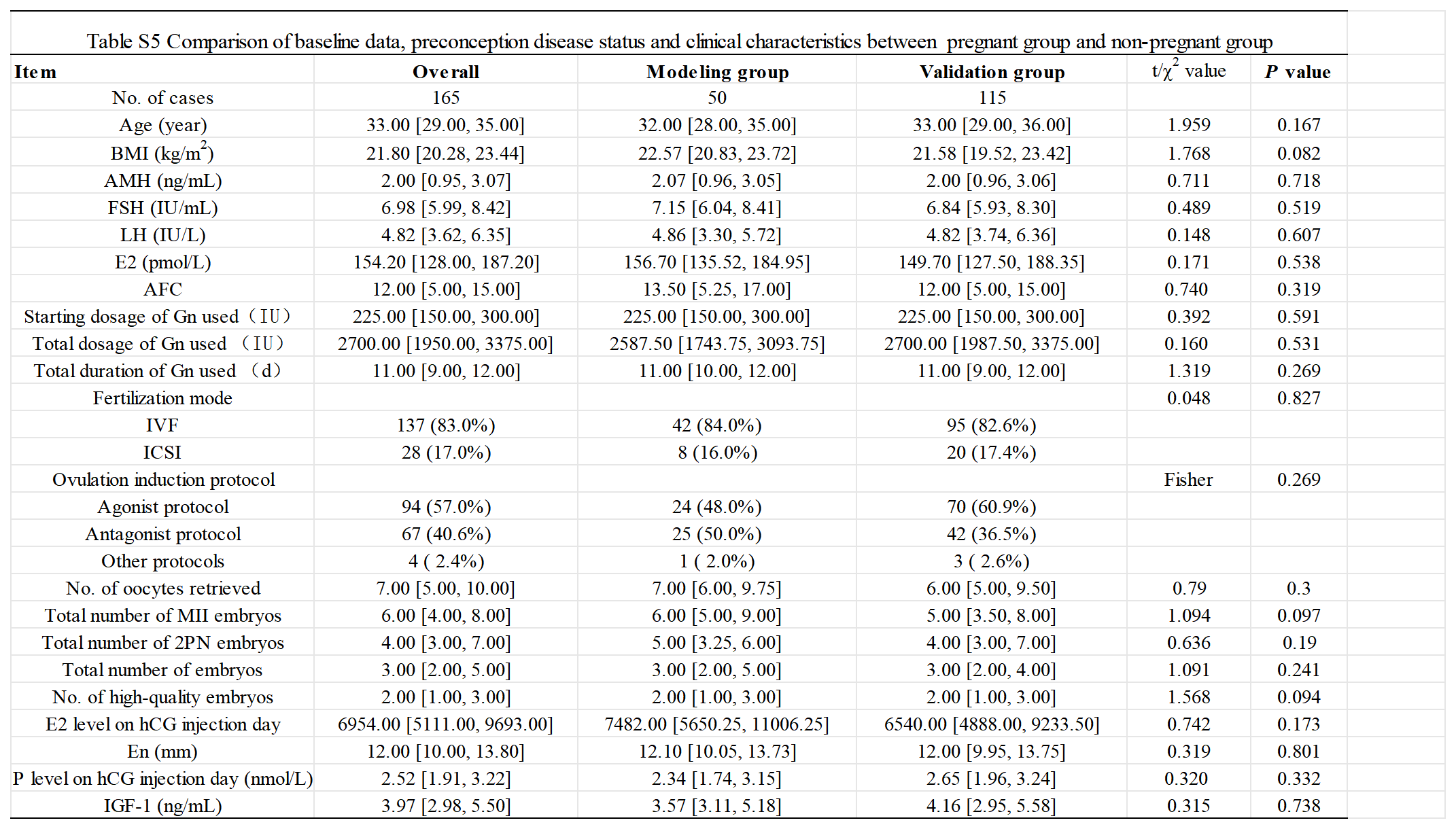


Note: BMI =body mass index; AMH=anti-Müllerian hormone; FSH=follicle-stimulating hormone; LH=luteinizing hormone; E_2_=estradiol; AFC=Antral follicle count at cycle entry; *P* level on hCG injection day =Progesterone level on hCG injection day (nmol/L).

**Table S6 Antibodies**

| **Antibodies** | **Vendors; Cat. No.** | **Source** | **Dilution/Applications** |
| --- | --- | --- | --- |
| IGF-1 | Abcam; ab106836 | Rabbit | 1:500 (WB) |
| IGF-1R | Beyotime Biotechnology; AF7182 | Rabbit | 1:500 (WB);  1:200 (IF) |
| β-actin | Proteintech; 60008-1-Ig | Mouse | 1: 5000 (WB) |
| CYP19A1 | Beyotime Biotechnology; AF6231 | Rabbit | 1: 200 (IF) |
| ERβ | PROTEINTECH; 14007-1-AP | Rabbit | 1: 200 (IF) |
| FSHR | PROTEINTECH; 22665-1-AP | Rabbit | 1: 200 (IF) |
| IGFALS | Biaworld; BS5607 | Rabbit | 1:1000 (WB);  1:300 (IF) |

## Table S7 Primers

| **Primer** | **Sequence** | **Application** |
| --- | --- | --- |
| *Igf-1r^fl/fl^-F* | CTTCCCAGCTTGCTACTCTAGG | Genotype |
| *Igf-1r^fl/fl^-R* | CAGGCTTGCAATGAGACATGGG | Genotype |
| *Foxl2-CreER^T2^-F* | ATGGACATGTTCAGGGATCG | Genotype |
| *Foxl2-CreER^T2^-R* | AGGGTGTTATAAGCAATCCCCAG | Genotype |
| *Igfals-F* | GATCCTTCTGGCTACCGTTTCTCTT | Genotype |
| *Igfals-R* | GGTCAAGGTGTCTGTCTTGTTTGGG | Genotype |
| *IGF-1-F-Human* | GCAATGGGAAAAATCAGCAGT | qPCR |
| *IGF-1-R-Human* | ACTGAAGAGCATCCACCAGC | qPCR |
| *IGF-1R-F-Human* | TGCCCGGAATTGCATGGTAG | qPCR |
| *IGF-1R-R-Human* | CCATGACGAAGCGAAGGACT | qPCR |
| *Inhbb-F-Mouse* | CTTCGTCTCTAATGAAGGCAACC | qPCR |
| *Inhbb-R-Mouse* | CTCCACCACATTCCACCTGTC | qPCR |
| *Inhba-F-Mouse* | TGAGAGGATTTCTGTTGGCAAG | qPCR |
| *Inhba-R-Mouse* | TGACATCGGGTCTCTTCTTCA | qPCR |
| *Ctgf-F-Mouse* | GGGCCTCTTCTGCGATTTC | qPCR |
| *Ctgf-R-Mouse* | ATCCAGGCAAGTGCATTGGTA | qPCR |
| *Cfh-F-Mouse* | AGGCTCGTGGTCAGAACAAC | qPCR |
| *Cfh-R-Mouse* | GTTAGACGCCACCCATTTTCC | qPCR |
| *Kctd14-F-Mouse* | CAAAGGTCATCTATGGAGCCAG | qPCR |
| *Kctd14-R-Mouse* | GAGAAGCCCAAAGTAGGTGCC | qPCR |
| *Gas6-F-Mouse* | TGCTGGCTTCCGAGTCTTC | qPCR |
| *Gas6-R-Mouse* | CGGGGTCGTTCTCGAACAC | qPCR |
| *Fshr-F-Mouse* | ACCATGGCTTAGAAAATCTGAAG | qPCR |
| *Fshr-R-Mouse* | GATCCCCAGGCTGAGTCATA | qPCR |
| *Cyp19a1-F-Mouse* | ATGTTCTTGGAAATGCTGAACCC | qPCR |
| *Cyp19a1-R-Mouse* | AGGACCTGGTATTGAAGACGAG | qPCR |
| *Cyp11a1-F-Mouse* | AGGTCCTTCAATGAGATCCCTT | qPCR |
| *Cyp11a1-R-Mouse* | TCCCTGTAAATGGGGCCATAC | qPCR |
| *Lhcgr-F-Mouse* | CGCCCGACTATCTCTCACCTA | qPCR |
| *Lhcgr-R-Mouse* | GACAGATTGAGGAGGTTGTCAAA | qPCR |
| *Esr1-F-Mouse* | AAAGGCGGCATACGGAAAGAC | qPCR |
| *Esr1-R-Mouse* | CTCCTGAAGCACCCATTTCAT | qPCR |
| *Esr2-F-Mouse* | CTGTGCCTCTTCTCACAAGGA | qPCR |
| *Esr2-R-Mouse* | TGCTCCAAGGGTAGGATGGAC | qPCR |
